# The Effectiveness of the BITSEA as a Tool to Early Detect Psychosocial Problems in Toddlers, a Cluster Randomized Trial

**DOI:** 10.1371/journal.pone.0136488

**Published:** 2015-09-18

**Authors:** Ingrid Kruizinga, Wilma Jansen, Nicolien C. van Sprang, Alice S. Carter, Hein Raat

**Affiliations:** 1 Department of Public Health, Erasmus University Medical Center, Rotterdam, the Netherlands; 2 Department of Youth Policy, Rotterdam Municipal Health Service (GGD Rotterdam- Rijnmond), Rotterdam, the Netherlands; 3 Public Health Care for Youth Rijnmond, Rotterdam, The Netherlands; 4 Department of Psychology, University of Massachusetts Boston, Boston, United States of America; National Institute of Child Health and Human Development, UNITED STATES

## Abstract

**Objective:**

Effective early detection tools are needed in child health care to detect psychosocial problems among young children. This study aimed to evaluate the effectiveness of the Brief Infant-Toddler Social and Emotional Assessment (BITSEA), in reducing psychosocial problems at one year follow-up, compared to care as usual.

**Method:**

Well-child centers in Rotterdam, the Netherlands, were allocated in a cluster randomized controlled trial to the intervention condition (BITSEA—15 centers), or to the control condition (‘care-as-usual’- 16 centers). Parents of 2610 2-year-old children (1,207 intervention; 1,403 control) provided informed consent and completed the baseline and 1-year follow-up questionnaire. Multilevel regression analyses were used to evaluate the effect of condition on psychosocial problems and health related quality of life (i.e. respectively Child Behavior Checklist and Infant-Toddler Quality of Life). The number of (pursuits of) referrals and acceptability of the BITSEA were also evaluated.

**Results:**

Children in the intervention condition scored more favourably on the CBCL at follow-up than children in the control condition: B = -2.43 (95% confidence interval [95%CI] = -3.53;-1.33 p<0.001). There were no differences between conditions regarding ITQOL. Child health professionals reported referring fewer children in the intervention condition (n = 56, 5.7%), compared to the control condition (n = 95, 7.9%; p<0.05). There was no intervention effect on parents’ reported number of referrals pursued. It took less time to complete (parents) or work with (child health professional) the BITSEA, compared to care as usual. In the control condition, 84.2% of the parents felt (very) well prepared for the well-child visit, compared to 77.9% in the intervention condition (p<0.001).

**Conclusion:**

The results support the use of the BITSEA as a tool for child health professionals in the early detection of psychosocial problems in 2-year-olds. We recommend future studies in large and varied populations to replicate these findings.

**Trial registration:**

Current Controlled Trials NTR2035

## Introduction

The prevalence of psychosocial problems, such as behavioural and emotional problems, is relatively high in preschool children [1–3]; child health professionals identify psychosocial problems in 7–25% of the preschool children [2–5]. It is important to detect problems at an early stage, since the identification and treatment of psychosocial problems at a young age may reduce problems and increases competencies at later ages [6,7]. In the Netherlands, the health care system offers publicly funded preventive programs for all children from birth to the age of 19 years. This setting offers an excellent opportunity to detect psychosocial problems early.

A feasible approach for facilitating early detection of psychosocial problems is to use parent-completed questionnaires as part of routine primary care visits (i.e. well-child visits) [8]. Early detection instruments for psychosocial problems, intended for use in preventive child health care, should have adequate psychometric properties, and should also be short, easy to administer, score and interpret [9,10]. Furthermore, early detection instruments that are used in a public health care setting should cover a broad range of psychosocial problems, since limited time and capacity for the well-child visits make it undesirable to screen for each psychosocial problem separately. Also, it has been shown that psychosocial problems tend to co-occur, [11,12] and that individual problems may apply to more than one disorder [13].

The Child Behavior Checklist 1.5–5 (CBCL1.5–5) [14] and Infant-Toddler Social and Emotional Assessment (ITSEA) [15,16] are early detection instruments that are well-validated and measure a broad range of psychosocial problems, and in the case of the ITSEA also delays in competencies. However both instruments are too extensive to apply in the context of well-child visits. Short comprehensive instruments that are appropriate to measure psychosocial problems in children of preschool age are limited [17]. Existing instruments, such as the Eyberg Child Behavior Inventory[18] or the Toddler Behavior Screening Inventory[19], only measure problem behaviour and do not address social-emotional competencies. Measuring delays in social-emotional competence, however, is also important since delays in competence are for instance related to internalising and externalising problems later in life[20]. There remains a need for a short instrument that measures both problems and delays in competence.

The Brief Infant-Toddler Social and Emotional Assessment (BITSEA) [21] is a short (42 items) questionnaire, that measures both problems (Problem scale) and delays in the acquisition of competencies (Competence scale) in 1–3 year olds, and also consists of items designed to measure symptoms of autism spectrum disorders. Several studies, conducted among a large (N = 3127) and diverse community sample in the preventive child health care of the Netherlands, have indicated that the psychometric properties of the BITSEA are acceptable to good [22]. An adequate Cronbach’s alpha (i.e. >0.70[23]) was found for the Problem scale (0.76) and marginal for the Competence scale (0.63). Test-retest reliability was adequate (>0.70 [24]) for the Problem scale (0.75) and marginal for the Competence scale (0.61). The BITSEA Problem scale was positively correlated with the CBCL, Pearson coefficients of 0.66 (Internalizing), 0.65 (Externalizing) and 0.75 (Total Problem). The BITSEA Competence score was negatively correlated with the CBCL, Pearson coefficients of -0.26 (Internalizing), -0.23 (Externalizing) and -0.26 (Total Problem). All correlations were significant (p<0.01). The mean BITSEA score was compared between a group of parents that worried about the development of their child and a group that did not worry. The Problem and Competence score were significantly less favourable in the group of parents that worried, compared to the group of parents that did not worry (effect sizes were respectively 0.93 and 0.52). Additionally, the BITSEA is able to discriminate between children with and without psychosocial problems; the Problem scale sensitivity is 0.83 and the specificity is 0.84; the Competence scale sensitivity is 0.95 and the specificity is 0.90 (with respectively reference groups of children with a CBCL Total Problem score > 60, and children diagnosed with autism spectrum disorder) [25,26]. These results are confirmed by studies in other countries [21,27–31].

### Objective of the study

In the present study, we evaluated the effectiveness of the BITSEA, a questionnaire that supports child health professionals in detecting psychosocial problems in 2-year olds, in psychosocial problems and health related quality of life at child age 3-years (at one year follow-up), compared to care as usual. In care as usual (i.e. control condition), the KIPPPI (KIPPPI is a Dutch acronym for Brief Instrument Psychological and Pedagogical Problem Inventory) is used [32].

The research questions in this study were:

1. Are there fewer parent reported psychosocial problems at follow-up (i.e. lower Child Behavior Checklist [CBCL] Total Problem score) in the intervention group, compared to care as usual?
2. Is health related quality of life at follow-up better (i.e. higher Infant-Toddler Quality of Life [ITQOL], Growth and Development, and General Health Perceptions scores) in the intervention group, compared to care as usual?

Additionally, we explored whether there is a difference between conditions in number of referrals and pursuits of referrals, and whether there is a difference between conditions in the acceptability as perceived by parents and child health professionals.

## Methods

### Ethics Statement

The Medical Ethical Committee of the Erasmus Medical Center has reviewed the study proposal and granted permission to conduct the study (MEC-2009-092, February 3^rd^ 2009). They decided that the study does not fall within the ambit of the Medical Research Involving Human Subjects Act (‘WMO’). All parents who participated in the study provided written informed consent. We are prepared to make the data available upon request.

### Study design

**Fig 1:**
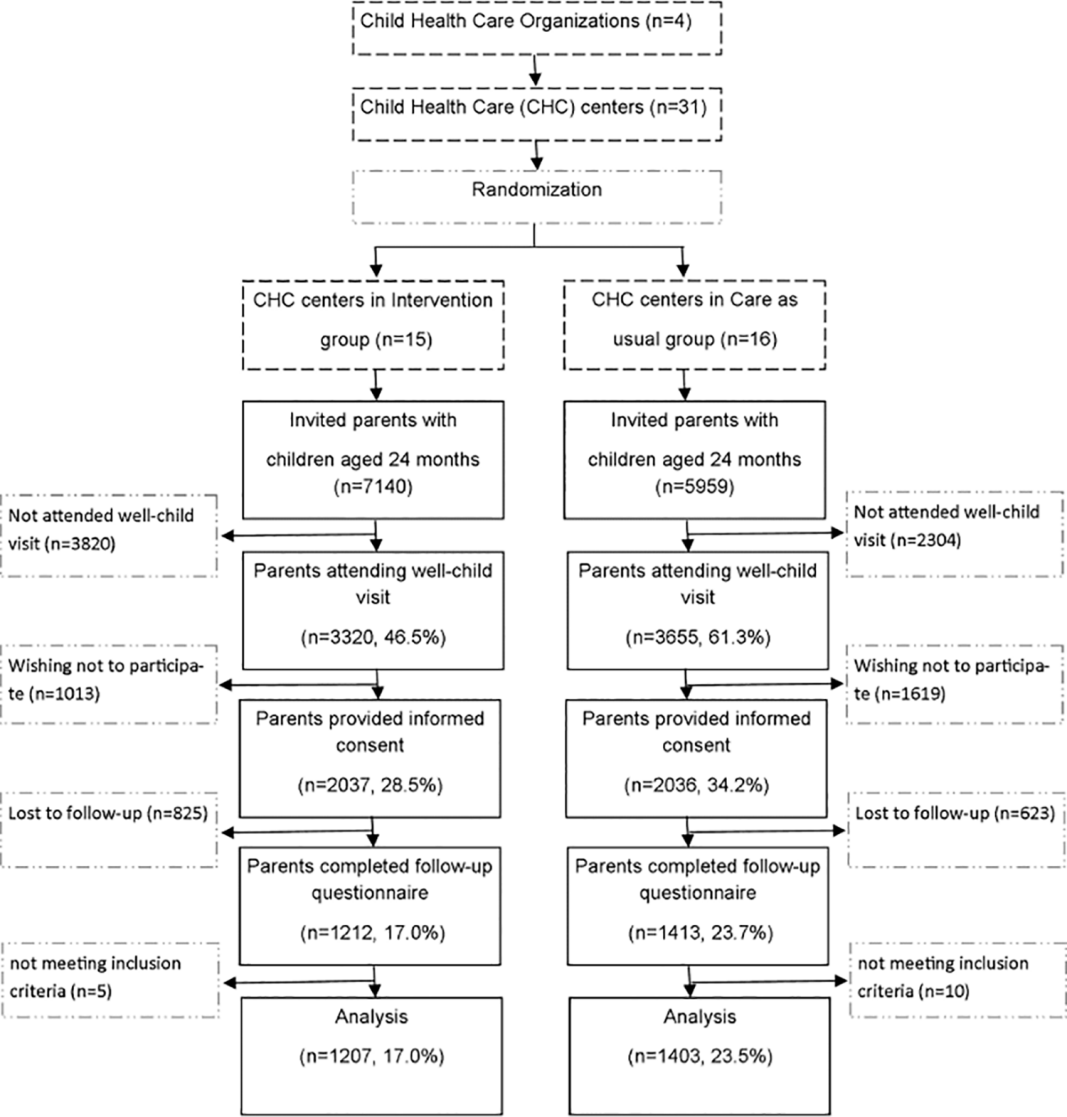
Flowchart of the participants through the study.

The present study is a cluster randomized controlled trial, conducted in child health care centers in the larger Rotterdam area in the period April 2010-April 2012. Details of our study design were published previously [33]. Child health care centers were randomly allocated (by researcher IK) to the control group (16 child health centers in total) or to the intervention group (15 child health centers in total), stratified by organization, using random numbers. See Fig 1.

At baseline, parents of children aged 24 months old were invited by child health care organizations for a well-child visit. Along with this invitation, parents received information regarding this study and a child health monitor questionnaire, including among others the CBCL and either the BITSEA or KIPPPI. The BITSEA or KIPPPI was used during the well-child visit by the child health professional to assess the psychosocial development of the child. Child health professionals were not blind to the answers on the CBCL, however they did not score the CBCL and they were instructed not to use the answers that parents provided on the CBCL in their assessment of the psychosocial development of the child. Prior to the start of the study, the child health professionals in the intervention group were trained in the use and scoring of the BITSEA. Instructions were provided by the researchers and a specialized psychiatrist.

At one-year follow-up (child aged 36 months), parents who had agreed to participate in the study and provided informed consent, were sent a follow-up questionnaire (with a maximum of 2 reminders), containing the CBCL, ITQOL, either the BITSEA or the KIPPPI and questions regarding pursuit of referrals in the previous year.

Blinding: It was not possible to conduct a double blind study; both parents and child health care providers were aware of the type of questionnaire that was used. Some child health professionals may have been aware that the BITSEA was recommended as ‘a promising questionnaire’ by an advisory committee to the Dutch government [34], which could have influenced their assessment of the child and subsequent (referral) decisions. Also, in this study, it was impossible to keep members of the study team blind.

Treatment contamination: It is not plausible that many parents were aware of the BITSEA being possibly better than care as usual, so we do not expect that being assigned to either condition influenced the responses given on the questionnaires. Also, it is not plausible that parents chose to go to another child health care center, outside of the neighborhood where they live, because they preferred the other instrument. For professionals in each of the condition groups it was not feasible to use the other instrument. So, the presence of treatment contamination was not likely.

### Study population

#### Child health care centers

Four child health care organizations consisting of 31 distinct child health centers (i.e. the clusters) that cover both urban and rural areas in the larger Rotterdam area, participated in this study. The characteristics of the clusters per condition are: Intervention condition: M_size_ = 78.0, mean SD_size_ = 55.29; M_CBCL_ = 17.8, mean SD_CBCL_ = 3.00; M_age_ = 23.7, mean SD_age_ = 0.24; mean percentage boys = 49.1; mean percentage native = 75.5. Control condition: M_size_ = 80.0, mean SD_size_ = 73.92; M_CBCL_ = 19.1 mean SD_CBCL_ = 2.37; M_age_ = 24.0 mean SD_age_ = 0.26; mean percentage boys = 51.2; mean percentage native = 79.4.

#### Children and their parents

For a flowchart of the participants through the trial, see Fig 1. Parents of 13,099 2-year old children were invited to participate in the study [7,140 (54.5%) intervention; 5,959 (45.5%) control]. Parents of 6975 (53.2%) children [3,320 (47.6%) intervention; 3,655 (52.4%) control] attended the well-child visit. Parents of 4,073 (31.1%) [2,037 (50.0%) intervention; 2,036 (50.0%) control] provided informed consent. Parents of 2,625 (20.0%) children [1,212 (46.2%) intervention; 1,413 (53.8%) control] also completed the follow-up questionnaire. Children were excluded from analyses if they had missing data on gender and ethnicity, and if there were too many missing items on the questionnaires (BITSEA Problem scale>5 items missing; BITSEA Competence scale>2 items missing; KIPPPI>25% items missing per scale). None of the children were under treatment of a mental health professional during the time of inclusion. After exclusion, 2,610 (64.1%) children [1,207 (59.5%) intervention; 1,403 (68.9%) control] remained for analyses.

### Power Calculation

As reported in the design paper on this study [33], power calculations indicated that, with 30 clusters, a total of 2100 children (and their parents) would be needed to detect a difference of 8 points on the CBCL1,5–5 between the control and intervention condition, assuming a standard deviation of the CBCL1 Total Problem score of 26.5 points [14] and an intra-cluster coefficient of 0.1, with a power of 0.80 and alpha 0.05

### Intervention condition

In the intervention condition, child health providers used the BITSEA completed by parents as an early detection tool for emotional and behavioural problems. The 42-item BITSEA is an early detection instrument for psychosocial problems and delays in competence, including autism spectrum disorders, in 12 to 36 month old children. Thirty-one items sum up to a Problem score (a high score is less favourable) and 11 items sum up to a Competence score (a low score is less favourable). Studies have shown that the BITSEA has good psychometric properties [21,22,27,29]. At baseline, child health providers calculated the Problem and Competence score of the BITSEA. To assess the psychosocial development of the child, cutpoints as identified in an earlier study [35] were used. Children with a BITSEA score above the cutpoint(s) warranted a conversation with the parents regarding the ‘at risk behaviour’, more specifically; level of worry, frequency and intensity of the behaviour, timing and onset of the behaviour, and the context in which the behaviour is manifested and the cultural meaning of the behaviour to the parent were discussed [35]. The child health professionals received additional training prior to the implementation of the BITSEA, so that they were able to objectively score the BITSEA and they had the appropriate skills to be able to have a conversation with the parents if an at risk score, whether on the Competence or Problem scale, on the BITSEA was present, in order to gain more insight in the significance of the child’s development. Protocols of the procedures that were applied and of training of the health care professionals will be made available upon request. The training was provided by the researchers and a child psychiatrist, and consisted of a presentation on psychosocial problems (prevalence, signals that indicate psychosocial problems, importance of early detection, worry of parents, conversation with parents during the well-child visit); background information of the BITSEA; and practical scoring instruction with the opportunity to practice. Also, the professionals received a manual and a scoring aid. The CHP made referrals to other health care providers in consultation with the parent when the ‘at risk behaviour’ seemed to be clinically significant. Follow up appointments were made if clarification of problems were necessary or for consultation on less serious problems that did not need referral.

### Control condition

In the control condition, care as usual was offered during the well-child visit, i.e. child health professionals used the parent completed KIPPPI as an early detection tool. The 67 item KIPPPI measures psychosocial problems, which might be possible pedagogical challenges for the parents. The KIPPPI consist of a Wellbeing scale (31 items, describing problems related to eating/drinking; sleeping; activity; mood; behaviour), Competence scale (25 items, describing problems related to cognitive development; language; play; contact), and an Autonomy scale (11 items, describing problems related to toilet training; motor skills; independence). The KIPPPI Total score is the sum of the scale scores (high scores on the KIPPPI are less favourable), and was calculated in a previous study to be able to evaluate the psychometric properties of the KIPPPI [36]. Studies have shown that the KIPPPI has adequate to good reliability and validity [36,37]. However, in care as usual the KIPPPI is not scored by the child health professional; the KIPPPI serves as a guide in the assessment of the development of the child and its use therefore relies on the subjective interpretation by the child health professional of the answers given on the KIPPPI. The reason that the KIPPPI was not scored in care as usual is that no empirically determined cutpoints exist. No additional training of the child health professionals was provided. At baseline, the child health professional discusses the items with high ratings (indicating a problem) with parents and assesses whether the difficulties stem from a problem in the child (i.e. psychosocial), or the parents (i.e. pedagogical), or the parent-child interaction. Based on this information the child health professionals may choose to refer parents and their child to other health care providers. Follow up appointments were made if clarification of problems were necessary or for consultation on less serious problems that did not need referral.

### Measures

#### Effect evaluation

The primary outcome measure in the present study is parent reported psychosocial problems of the children participating in the study at one year follow-up. In both the intervention and control condition, parents completed the CBCL1.5–5 at baseline and at follow-up. The well-validated [14] 100-item CBCL is designed for children aged 18-months to 5-years and has two domains (Internalizing and Externalizing) and provides a Total Problem score. A higher CBCL Total Problem score is less favourable. In this study, the Total Problem score (raw score) is used to measure psychosocial problems.

The secondary outcome measure in the present study is parent reported health related quality of life of the children participating in the study at one year follow-up. In both intervention and control condition, parents completed two scales of the ITQOL; Growth and Development, and General Health Perceptions. The ITQOL measures the health related quality of life of children between 2 months and 5 years old. [38,39]. Items were reversed scored (if appropriate) so that a higher score is more favourable.

#### Referrals and acceptability

In addition to the evaluation of the effect on parent reported psychosocial problems and health related quality of life, we also evaluated referrals and acceptability. Referrals were evaluated as a possible explanation for when a difference in outcome variables was found between conditions, and was defined as an actual referral to a mental health professional and/or the advice for a follow-up consult. Referrals were evaluated as registered by the child health professional at baseline on a separate registration form or in a digital medical record system depending on the automatization level of the child health organization involved, Additionally, the extent to which parents pursued received referrals was evaluated by parental report at follow-up.

Acceptability was evaluated from two perspectives; 1) parents evaluated at baseline the child health monitor questionnaire that included either the BITSEA or KIPPPI on the following aspects: a) perceived duration (dichotomized as ‘(much) too short/exactly good-(much) too long’); b) actual duration (dichotomized as ‘shorter than 15 minutes-longer than 15 minutes’); c) preparation for the well-child visit (dichotomized as ‘not good (at all)/mediocre-(very) good’); and 2) 105 (92.9%) child health professionals completed, about a half year after the start of the study, an electronic questionnaire regarding the perceived acceptability of the questionnaires. The following aspects were evaluated: a) time spent on the questionnaire before the well-child visit (i.e. scoring of the BITSEA or looking at the answers on the KIPPPI); b) time spent on the questionnaire during the well-child visit (i.e. discussing the scores/answers with the parents; c) supportive in detecting psychosocial problems (binary; yes or no); d) supportive in the conversation with the parents (binary; yes or no); e) supportive in the assessment of the development of the child (binary; yes or no).

#### Demographic variables

Demographic variables were assessed in the baseline questionnaire: parental age, country of birth, and educational level and child gender and ethnicity. A child was considered native if both parents were born in the Netherlands, according to Statistics Netherlands [40].

### Analyses

For the demographic characteristics and for baseline CBCL Total Problem score we tested whether there was a difference between parents and children who remained in the study, and parents and children who were lost by follow-up (i.e. drop-out). We used an independent t-test for continuous variables and Chi-square for categorical variables. Cohen’s d was calculated to evaluate effect sizes. Furthermore, we tested with logistic regression analysis whether conditions differed in the characteristics of the drop-out group. Odds ratios were calculated for missingness, with condition, baseline CBCL Total Problem score and the above mentioned variables as predictors.

Descriptive statistics were used to describe the characteristics of the parents and children in the two conditions. Differences between the intervention and control condition, as measured at baseline, were tested with an independent t-test (continuous variables) and Chi-square test (categorical variables). Effect sizes of the differences between intervention and control condition were calculated. For the continuous variables Cohen’s d was calculated: *Cohen’s d = [mean1–mean2]/SD1* and is interpreted as follows: 0.20≤*d*<0.50 indicates a small effect, 0.50≤*d*<0.80 indicates a medium effect and *d*≥0.80 indicates a large effect. [41] For the categorical variables Cramers V [42] was calculated: V = (chi^2^/(sample size(smallest value of number of columns or rows)))^1/2^. For the interpretation of Cramers V, the following characterization is applied <0.10 low association; 0.10–0.25 moderate association; >0.25 high association;

#### Effect evaluation

The multilevel regression analysis (with child health centers as clusters) is performed with psychosocial problems at follow-up as primary outcome measure (i.e. CBCL Total Problem score at follow-up) and with health related quality of life at follow-up as secondary outcome measure (i.e. ITQOL Growth and Development scale, and General Health Perceptions scale), and the intervention condition as independent variable (i.e. BITSEA or KIPPPI). The multilevel regression analyses were adjusted for psychosocial problems at baseline (i.e. CBCL Total Problem score at baseline), child gender and ethnicity, because previous studies show that gender and ethnicity are associated with psychosocial problems [43–45]. Additionally, when a significant intervention effect was found, the analyses were with correction for all variables that differed between conditions at baseline. Interaction effects for child gender and condition, and child ethnicity and condition on psychosocial problems and health related quality of life were explored when the main effect of condition was significant.

The intracluster correlation coefficient (ICC) is calculated as ρ = S^2^_b_/(S^2^_b_+S^2^_w_), where S^2^_b_ is the variance between the cluster and S^2^_w_ is the variance within clusters. An ICC of less than 0.1 is considered small. [46] The ICC in this study is; ρ = 3.2597/(254.41+3.2597) = 0.01.

#### Referrals and acceptability

Differences between the intervention and control condition in number of referrals, pursuits of referrals and acceptability were tested with an independent t-test (continuous variables) and Chi-square test (categorical variables). When these tests indicated a significant difference between conditions, an odds ratio was calculated.

Multilevel regression analysis were performed in SAS 9.3 (SAS Institute Inc., 2011), all other analyses were performed in SPSS 21.0 (SPSS Inc., 2012).

## Results

### Study sample

Drop out was for the intervention and control condition, respectively 59.2% and 68.9% of the parents who attended the well-child visit and provided informed consent. Drop-out was higher among younger parents with a lower education, parents and children with a non-native Dutch ethnicity and older children (measured at baseline, p<0.05). However, effect sizes (Cohen’s d and Cramer’s V) for these differences were low and ranged from 0.01–0.22. There was no difference between children included in the study and drop-out in gender and CBCL Total Problem score at baseline. Missing value analysis revealed there was no difference in drop-out between the conditions. See Fig 1 for a flow-chart of the participants through the study. No data was available on the group of parents who did not attend the well-child visit. Therefore, evaluating the differences between parents included in the study and parents who did not attend the well-child visit (and thus not participated in the study), was not possible.

Demographic characteristics of the parents and children are presented in Table 1, as well as the mean questionnaire scores at baseline. Participants in the intervention condition differed (p<0.05) in the following aspects from the control condition (see Table 1), mean age of the mother and father was higher (effect size is for both parents 0.09); fewer mothers and fathers were born in the Netherlands (effect size is respectively 0.12 and 0.14); more mothers and fathers had attended vocational education or university (effect size is respectively 0.10 and 0.12). Compared to the control condition, the intervention condition consisted of fewer native Dutch children (effect size = 0.13). Children in the intervention condition were younger (effect size = 0.27) and scored less favourably on the CBCL Total Problem score at baseline (effect size = 0.09). Effect sizes of all differences in variables between intervention and control condition were small [41]. We have performed the analyses corrected for all the variables that differed between intervention and control condition, and the results were similar as those reported (see S1 Table).

**Table 1:** Baseline demographic characteristics and mean questionnaire scores for the intervention and control condition, N = 2610.

	Intervention condition	Control condition	
	BITSEA, n = 1207	KIPPPI, n = 1403	effect size
**Mother characteristics**			
Mean (SD) age	34.29 (4.69)*	33.88 (4.67)*	0.09^a^
Country of birth [n(%) Dutch]	920 (76.22)**	1198 (85.39)**	0.12^b^
Educational level [n(%) higher vocational/university]	631 (54.30)**	592 (43.98)**	0.10^b^
**Father characteristics**			
Mean (SD) age	36.80 (5.29)*	36.35 (5.15)*	0.09^a^
Country of birth [n(%) Dutch]	892 (73.90)**	1199 (85.46)**	0.14^b^
Educational level [n(%) higher vocational/university]	571 (51.63)**	524 (40.03)**	0.12^b^
**Child characteristics**			
Gender [n(%) boys]	594 (49.20)	733 (52.20)	n.a.
Ethnicity [n(%) native Dutch]	849 (70.34)**	1139 (81.18)**	0.13^b^
Mean (SD) age	23.69 (0.69)**	23.98 (1.09)**	0.27^a^
Mean (SD) CBCL Total Problem score	18.87 (15.31)*	20.32 (15.66)*	0.09^a^
Mean (SD) BITSEA Problem score	7.38 (4.85)	n.a.	n.a.
Mean (SD) BITSEA Competence score	17.90 (2.93)	n.a.	n.a.
Mean (SD) KIPPPI score	n.a.	40.93 (14.16)	n.a.

* significant difference, p<0.05.

** significant difference, p<0.01.

^a^ Cohen's d (Mean1-Mean2/SD1), is considered small.

^b^ Coefficient Phi, is considered small.

### Effect evaluation

#### Psychosocial Problems

As presented in Table 2, children in the intervention condition had lower mean CBCL Total Problem scores compared to children in the control condition both at baseline (respectively M = 18.87, SD = 15.31 and M = 20.32, SD = 15.66) and follow-up (respectively M = 19.32, SD = 15.71 and M = 22.51, SD = 16.28). In the control condition, mean CBCL Total Problem score was significantly higher at follow-up than at baseline. There was no difference in the intervention condition between baseline and follow-up CBCL Total Problem score (p>0.05).

**Table 2:** Mean (SD) CBCL Total Problem score, and ITQOL Growth and Development, and General Health scores (n = 2610).

	Intervention, n = 1207		Control, n = 1403		Difference between conditions
	Baseline	Follow-up	Baseline	Follow-up	Effect size* (baseline;follow-up)
CBCL Total Problem score	18.87 (15.31)^a^	19.32 (15.71)^a^	20.32 (15.66)^a^ ^b^	22.51 (16.28)^a^ ^b^	0.09;0.20
ITQOL Growth and Development	n.a.	91.39 (11.62)	n.a.	92.11 (10.09)	n.a.;0.07
ITQOL General Health	n.a.	81.77 (14.66)	n.a.	82.43 (14.02)	n.a.;0.05

* Cohen's d (Mean1-Mean2/SD1), is considered small.

^a^ significant difference between intervention and control condition in CBLC Total Problem score at baseline and follow-up (p<0.05).

^b^ significant difference within control condition between baseline and follow-up CBCL Total Problem score (p<0.05).

Children in the intervention condition scored more favourably on the CBCL Total Problem score at follow-up than children in the control condition (controlled for CBCL Total Problem score at baseline, child gender and ethnicity); the unstandardized regression coefficient for type of condition was significant (p<0.001), B = -2.43 (95% confidence interval [95%CI] = -3.53;-1.33), see Table 3. Effect sizes were small: Cohen’s D for the difference between conditions in CBCL Total Problem score was at baseline 0.09 and at follow-up 0.20, see Table 2.

**Table 3:** Regression coefficients and confidence intervals (95% CI) from the multilevel regression models evaluating the association between condition and CBCL Total Problem score and ITQOL scores at follow-up corrected for confounders (n = 2230).

	Model 1	Model 2	Model 3	Model 4	Model 5
	beta (95%CI)	beta (95%CI)	beta (95%CI)	beta (95%CI)	beta (95%CI)
**Primary outcome measure: CBCL Total Problem score at follow-up**					
Condition (intervention)	-3.45 (-4.80;-2.10)***	-2.40 (-3.71;-1.09)***	-2.43 (-3.53;-1.33)***	-2.28 (-3.77;-0.78)**	-5.07 (-7.22;-2.92)***
CBCL Total Problem score baseline		0.72 (0.68;0.76)***	0.71 (0.67;0.75)***	0.71 (0.67;0.74)***	0.71 (0.67;0.74)***
Child gender (boy)			1.47 (0.47;2.47)**	1.60 (0.29;2.91)*	1.47 (0.48;2.47)**
Child ethnicity (native)			-0.96 (-2.19;0.27)	-0.96 (-2.19;0.26)	-2.67 (-4.37;-0.96)**
Condition x gender				-0.31 (-2.33;1.70)	
Condition x ethnicity					3.44 (1.05;5.83)
**Secondary outcome measure: ITQOL Growth and Development score at follow-up**					
Condition (intervention)	-0.66 (-1.58;0.26)	-0.76 (-1.66;0.14)	-0.76 (-1.68;0.16)		
CBCL Total Problem score baseline		-0.12 (-0.16;-0.08)***	-0.12 (-0.16;-0.08)***		
Child gender (boy)			-0.62 (-1.52;0.28)		
Child etnicity (native)			-0.16 (-1.26;0.94)		
**Secondary outcome measure: ITQOL General Health score at follow-up**					
Condition (intervention)	-0.70 (-2.05;0.65)	-1.01 (-2.24;0.22)	-0.10 (-1.32;1.12)		
CBCL Total Problem score baseline		-0.23 (-0.27;-0.19)***	-0.23 (-0.27;-0.19)***		
Child gender (boy)			-1.43 (-2.59;-0.27)*		
Child etnicity (native)			0.81 (-0.60;2.22)		

*** p<0.001

** p<0.01

* p<0.05.

Similar results were found when the analyses were performed corrected for all the variables that differed between conditions at baseline.

### Health related quality of life

There were no significant differences (p>0.05) between conditions in ITQOL scores on the Growth and Development scale nor on the General Health Perceptions scale, see Table 3.

### Interactions

Interaction effects were explored. For the primary outcome variable (i.e. CBCL Total Problem score), we evaluated separately condition by gender and condition by ethnicity interactions; both interaction effects were non-significant (p>0.05) (see Table 3). For the secondary outcome variable (i.e. ITQOL score on the Growth and Development scale and General Health scale), we did not evaluated interaction effect since the main effect of condition was not significant (p>0.05).

### Referrals and acceptability

#### Referrals

Given the differences between conditions on the primary outcome, we evaluated the number of referrals as a possible explanation. There was a main effect of the intervention (p = 0.042) on the number of referrals registered by the child health professionals: fewer children in the intervention condition (n = 56, 5.7%) were referred, compared to the control condition (n = 95, 7.9%). Children in the control condition were 1.42 times more likely to be referred (odds ratio = 1.42, 95% CI = 1.01–2.00), compared to children in the intervention condition. There was no effect of the intervention (p>0.05) on the number of parent reported referrals pursued (n = 58, 92.1% versus n = 54, 91.5%, for the intervention and control groups respectively).

#### Acceptability

There were significant differences (p<0.001) between the intervention and control condition in the acceptability as perceived by parents. Completion of the BITSEA took less time compared to completion of the KIPPPI: The duration of completion was less than 15 minutes for 592 parents (49.9%) in the intervention condition and for 555 parents (40.6%) in the control condition. The preparation for the well-child visit was considered (very) good by 908 parents (77.9%) in the intervention condition and by 1146 parents (84.2%) in the control condition.

The only significant difference (p<0.01) between intervention and control condition in acceptability, as perceived by the child health professionals, was the time that they spent on the questionnaire during the well-child visit. In the intervention condition the mean time was 8.22 minutes (SD = 5.59) and in the control condition 12.08 minutes (SD = 6.16). See Table 4.

**Table 4:** Differences between intervention and control condition regarding acceptability.

	Intervention	Control
	BITSEA	KIPPPI
**Acceptability parents—N = 2610**		
Perceived duration—(too)short/exactly good [n(%)]	820 (69.7)	924 (67.6)
Actual duration—< 15 minutes [n(%)]	592 (49.9)***	555 (40.6)***
Preparation for well-child visit—(very) good preparation [n(%)]	908 (77.9)***	1146 (84.2)***
**Acceptability Child health professional—n = 105**		
Time spend before consult [M (SD)]	4.85 (3.69)	3.95 (2.44)
Time spend during consult [M (SD)]	8.22 (5.89)**	12.08 (6.16)**
Supportive detecting problems [n(%)]	31 (72.1)	31 (64.6)
Supportive conversation with parents [n(%)]	29 (67.4)	39 (83.0)
Supportive assessment development [n(%)]	25 (62.5)	20 (43.5)

*** significant difference, p<0.001

** significant difference, p<0.01

* significant difference, p<0.05.

## Discussion

In the present study, we evaluated the effect at one year follow-up of the use by preventive child health care of the BITSEA as a questionnaire for the early detection of parent reported psychosocial problems in 2-year olds, compared to care as usual; the KIPPPI. Furthermore, we assessed the number of referrals, number of pursuits of referrals and acceptability of the BITSEA and KIPPPI as perceived by parents and professionals. The results indicate that children in the intervention condition had fewer psychosocial problems at follow-up (i.e. lower CBCL Total Problem score), compared to the control condition. No intervention effects were observed regarding health related quality of life. Child health professionals registered more referrals in the control condition than in the intervention condition. The BITSEA takes less time for parents to complete and for the child health professional to score, compared to the KIPPPI. Parents perceived the KIPPPI as a better preparation for the well-child visit, compared to the BITSEA.

### Effect evaluation and referrals

The difference in CBCL Total Problem score between conditions at follow-up is significant, but the effect size is small. A large effect might not be expected given the relatively long follow-up period of one year in which many variables can influence the psychosocial well-being of a child.

One might expect that children who are referred will have fewer psychosocial problems one year later, due to more timely and appropriate care. However, this assumption is not supported by our results; in the intervention condition (BITSEA) there were relatively fewer referrals, but better psychosocial health at follow-up, compared to the control condition (KIPPPI). Noteworthy is the fact that children in the control condition had a significantly higher CBCL Total Problem score at follow-up compared to baseline, despite the fact that more children in the control condition were referred. In the intervention condition there was no difference between baseline and follow-up CBCL Total Problem score.

Secondary analyses were performed in an attempt to explain the primary results. First of all, we evaluated the differences (if any) in the mean CBCL subscale scores (i.e. Internalizing and Externalizing) between conditions in the total population and in the subgroup of referred children. The BITSEA and KIPPPI differ in content, so possibly children, either or not referred, might show different types of problems reflected in differences in baseline CBCL subscale scores. For the total population we found a difference between conditions on the CBCL Internalizing subscale (p = 0.048). Children in the KIPPPI group scored less favourably, compared to the BITSEA group. In the subgroup of referred children there was however no difference in CBCL subscale scores between conditions. This might indicate that internalizing problems in the KIPPPI group were less well detected, compared to the BITSEA group. This in turn might explain better outcomes at follow-up in the BITSEA group.

Second of all in two subgroups, 1) children with a ‘normal’ baseline CBCL Total Problem score and 2) children with an ‘at risk’(i.e. >60) baseline CBCL Total Problem scale, we tested whether the conditions had a different rate of referral (see S2 Table). This might give insight in the extent of over and under referral between conditions. The percentage of referrals in children with an ‘at risk’ or ‘normal’ score did not differ between conditions (p>0.05). We found an overall difference in number of referrals between conditions, but not when evaluated separately for ‘at risk’ and ‘low scores’, this might be explained by the relatively small size of the subgroups in these analyses.

At follow-up the children in the BITSEA group had the same CBCL Total Problem score as at baseline. However, children in the KIPPPI group had a less favourable CBCL Total Problem score at follow-up, compared to baseline. BITSEA therefore seems to add to prevent deterioration of psychosocial problems in 2-year-olds.

### Acceptability

Even though the BITSEA was scored, and the KIPPPI not, it took less time for parents and child health professionals to work with the BITSEA. Parents felt better prepared for the well-child visit after completing the KIPPPI, compared to the BITSEA. This might be, because the items of the KIPPPI are very structured and clustered per ‘domain’ (e.g. sleeping, eating, behaviour), whereas in the BITSEA items of the Problem and Competence scale are randomly arranged. This random arrangement might however contribute to less socially desirable answers, and therefore better validity.

### Limitations and strengths

Our study has several limitations. First, we recognize that there are different ways in which psychosocial problems can be measured. We measured the primary outcome, psychosocial problems, with the CBCL. As stated in the introduction, the CBCL is a well-validated questionnaire, however it is a parent-completed instrument, rather than direct assessment. The report by parents introduces the proxy-problem; self-report by two-year-old children on their psychosocial problems is not possible, because children of this age lack the necessary language skills and the cognitive abilities to interpret the questions and they do not have a long-term view of events.[47] Therefore, proxy by parents may be a useful alternative.[48] Direct assessment of psychosocial problems, might have been an alternative for parent reported measurements. Direct assessment, for example by a psychologist, of every child in this study was not feasible with our large sample size. Furthermore, our data on the number of referrals was unable to provide a conclusive explanation for the difference between conditions in psychosocial problems at one year follow-up. Although child health professionals were instructed not to look at the CBCL answers, 11.4% of the child health professionals indicated that sometimes they did. The child health professional did not have the CBCL score, so referral decision could not be influenced by the CBCL score, but the answers might have influenced their assessment of the child. More elaborate data on referral decision could provide more insight in this underlying mechanism.

Second, as reported in the publication of the study protocol [33], at follow-up we measured health related quality of life (i.e. General Health Perceptions subscale and the Growth and Development subscale of the Infant Toddler Quality of Life questionnaire [ITQOL]) [39,49]. This variable was not measured at baseline, due to restrictions on the length of the questionnaire booklet at baseline. The child health centers were randomly allocated to the intervention and control condition, therefore similar health related quality of life in the conditions at baseline may be assumed. Therefore, despite the absence of longitudinal data on the ITQOL we evaluated the difference in health related quality of life between the control and intervention condition. We analyzed the difference between conditions uncorrected, as well as corrected for CBCL Total Problem score at baseline, and additionally for CBCL Total Problem score, gender and ethnicity of the child. We found no differences between conditions (data not shown).

Third, parents evaluated not the acceptability of the BITSEA and KIPPPI specifically, but the entire child health monitor questionnaire. However, the only differences between these booklets, was the inclusion of either the BITSEA or the KIPPPI, so any differences between conditions may be attributed to these questionnaires.

Fourth, total drop-out rate was 35.9% (i.e. lost to follow-up). To provide a frame of reference regarding drop-out rates in studies conducted in the Dutch (preventive) child health care setting, we compared our drop-out rate to that in others studies; 37.1% (child obesity, with a 2 year follow-up period [50]); 21.7% (reduction of sugar-sweetened beverages, with a 1 year follow-up period [51]); and 6.6% (parent’s child safety behaviours, with a 6 month follow-up period [52]). The drop-out rate in our study is somewhat high, compared to other studies, but not uncommon in these types of studies. There were differences between intervention and control condition in drop-outs (respectively 40.5% and 30.6%). However, there was no difference between conditions in characteristics of people who were and were not retained in the study. So drop-out seemed to be non-selective, based on the variables we measured in this study. However, drop-out might be influenced by variables which we did not measure, such as migration rate. No information is available on the non-response group (i.e. parents that did not attend the well-child visit.) It might be possible that parents avoid attending the well-child visit because they are afraid of possible interventions from Youth Care, but it might also be possible that parents do not find it necessary to attend the well-child visit because they feel confident that their child has no problems.

Fifth, the size and other characteristics of the clusters (i.e. child health care centers) varied in both the intervention and the control condition. Overall, differences between the intervention and control group were relatively small. But the differences between the clusters, especially the size (i.e. number of children in each cluster), may have lowered the power of the study. However, the power of this study was relatively large. No data, on cluster level, was available on deprivation indices, so we were unable to evaluate whether an unequal distribution between intervention and control clusters, with regard to the level of deprivation in the catchment area, had impact on the results of this study.

Lastly, the group of child health professionals who evaluated the acceptability of the BITSEA was rather small (n = 105), however the response rate was very high (i.e. 92.2% of all child health professionals). The results reflect the situation in Rotterdam well, however generalizations to other areas or situations should be done with care.

Our study also has many strengths. First, our study design; random allocation of the well-child centers to the intervention and control condition eliminated systematic differences between these groups, which adds to the power of the study. Moreover, our longitudinal data made it possible to evaluate the impact of the early detection instrument under study over a longer period of time. Second, a major strength is that analyses were performed on a large and diverse community sample, which adds to the power of the results. Third, the questionnaires were applied in preventive child health care where they were used for the early detection of psychosocial problems. As the results suggests, it might be possible that child health care professionals do not always act solely on the BITSEA cutpoints if their professional expertise made them decide otherwise. See also the BITSEA manual [35], in which is stated that the following things should also be taken into account while assessing the development of the child: level of worry of the parents; frequency or intensity of the behaviour; timing or onset of the behaviour; context of the behaviour and the meaning of the behaviour in the cultural context of the parent. So the results reflect the outcomes as they will be in implemented, not just in theory.

### Future research

We recommend that future studies evaluate more elaborately which children are being referred and follow these children more closely over a longer period of time. Additionally, the application of cut-points by the child health professional should be evaluated in more detail: to what extent are other factors (such as parental worry, situational or cultural context), important in the decision to refer? We recommend future studies to evaluate the relationships between early detection, number and appropriateness of referrals, appropriateness of treatment, and health at follow-up.

This study was embedded within the Dutch system of preventive child health care provided by well-child centers in Rotterdam, the Netherlands. This may have consequences for the generalizability of our results in other areas and countries. Therefore, replication of our study in other, varied populations is recommended to confirm or reject the results presented in this study and to further explore pathways that explain the results of the present study.

## Conclusion

The results support the use of the BITSEA as a tool for child health professionals in the early detection of psychosocial problem in 2-year-olds. It appeared that children experience fewer psychosocial problems when the BITSEA was used as compared to care as usual at one-year follow-up. The difference in psychosocial problems between BITSEA and ‘care as usual’ group at follow-up could not be explained by a difference in number of referrals. In this study we focused on the questionnaires, however other elements that differed between conditions (such as scoring of the questionnaire and the training), might also be responsible for the difference between conditions. We recommend future studies in large and varied populations to replicate these findings and to explore the effective elements of the use of the questionnaires.

## Supporting Information

S1 CONSORT Checklist Consort Checklist. (PDF)

S1 Protocol Research Protocol. (PDF)

S1 Table Regression coefficients and confidence intervals (95% CI) from the multilevel regression models evaluating the association between condition and CBCL Total Problem score at follow-up corrected for confounders (n = 2230). (PDF)

S2 Table Number (percentages) of referred children in the total sample and in the subsample with baseline 'at risk' scores on the CBCL and BITSEA. (PDF)
