# Supplementary material for: The Effectiveness of the BITSEA as a Tool to Early Detect Psychosocial Problems in Toddlers, a Cluster Randomized Trial
Source: PLoS One. 2015 Sep 18;10(9):e0136488. doi: 10.1371/journal.pone.0136488 (PMC4575038; doi:10.1371/journal.pone.0136488)
Supplement: S1 CONSORT Checklist — (PDF) [file pone.0136488.s001.pdf]

CONSORT 2010 checklist of information to include when reporting a randomised trial*

Section/Topic	Item No	Checklist item	Reported on page No
**Title and abstract**			
	1a	Identification as a randomised trial in the title	See Title, p.1
	1b	Structured summary of trial design, methods, results, and conclusions (for specific guidance see CONSORT for abstracts)	See Abstract, p2
**Introduction**			
Background and objectives	2a	Scientific background and explanation of rationale	See Introduction, 1st, 2nd and 3rd paragraph, p.4-5
	2b	Specific objectives or hypotheses	See Introduction, 5th paragraph, p.5
**Methods**			
Trial design	3a	Description of trial design (such as parallel, factorial) including allocation ratio	See Methods section, 2nd paragraph ('Study design'), p.6-7
	3b	Important changes to methods after trial commencement (such as eligibility criteria), with reasons	Not applicable
Participants	4a	Eligibility criteria for participants	See Methods section, 4th paragraph ('Children and their parents'), p.7-8

	4b	Settings and locations where the data were collected	See Methods section, 3th paragraph ('child health care centers'), p.7
Interventions	5	The interventions for each group with sufficient details to allow replication, including how and when they were actually administered	See Methods section, 5th paragraph ('Intervention condition' and 'control condition'), p.8-9
Outcomes	6a	Completely defined pre-specified primary and secondary outcome measures, including how and when they were assessed	See Methods section, 7th paragraph ('Measures' subheading 'Effect evaluation'), p.10
	6b	Any changes to trial outcomes after the trial commenced, with reasons	Not applicable
Sample size	7a	How sample size was determined	Determination of sample size was reported in design paper
	7b	When applicable, explanation of any interim analyses and stopping guidelines	Not applicable
Randomisation:			
Sequence generation	8a	Method used to generate the random allocation sequence	See Methods section, 1st paragraph

			('study design'), p.6
	8b	Type of randomisation; details of any restriction (such as blocking and block size)	See Methods section, 1st paragraph ('study design'), p.6
Allocation concealment mechanism	9	Mechanism used to implement the random allocation sequence (such as sequentially numbered containers), describing any steps taken to conceal the sequence until interventions were assigned	See Methods section, 1st paragraph ('study design'), p.6
Implementation	10	Who generated the random allocation sequence, who enrolled participants, and who assigned participants to interventions	See Methods section, 1st paragraph ('study design'), p.6
Blinding	11a	If done, who was blinded after assignment to interventions (for example, participants, care providers, those assessing outcomes) and how	Not applicable
	11b	If relevant, description of the similarity of interventions	Not applicable
Statistical methods	12a	Statistical methods used to compare groups for primary and secondary outcomes	See Methods section, 8th paragraph ('Analyses', subheading 'Effect evaluation'), 12-13
	12b	Methods for additional analyses, such as subgroup analyses and adjusted analyses	See Methods section, 8th paragraph ('Analyses', subheading

			‘referrals and acceptability’), p.13
**Results**			
Participant flow (a diagram is strongly recommended)	13a	For each group, the numbers of participants who were randomly assigned, received intended treatment, and were analysed for the primary outcome	See Methods section, 4th paragraph (‘Children and their parents’), p.8 and Figure 1
	13b	For each group, losses and exclusions after randomisation, together with reasons	Figure 1
Recruitment	14a	Dates defining the periods of recruitment and follow-up	See Methods section, 1st paragraph (‘Study design’), p.6
	14b	Why the trial ended or was stopped	See Methods section, 1st paragraph (‘Study design’), p.6
Baseline data	15	A table showing baseline demographic and clinical characteristics for each group	Table 1, p.29
Numbers analysed	16	For each group, number of participants (denominator) included in each analysis and whether the analysis was by original assigned groups	Table 2-4, S1, p. 29-33
Outcomes and estimation	17a	For each primary and secondary outcome, results for each group, and the estimated effect size and its precision (such as 95% confidence interval)	Table 3, p.31
	17b	For binary outcomes, presentation of both absolute and relative effect sizes is recommended	Not applicable
Ancillary analyses	18	Results of any other analyses performed, including subgroup analyses and adjusted analyses, distinguishing pre-specified from exploratory	Table 4, S1, p.32-33
Harms	19	All important harms or unintended effects in each group (for specific guidance see CONSORT for harms)	Not applicable
**Discussion**			
Limitations	20	Trial limitations, addressing sources of potential bias, imprecision, and, if relevant, multiplicity of analyses	See

			Discussion section, 4th paragraph (‘Limitations and strengths’), p.18-20
Generalisability	21	Generalisability (external validity, applicability) of the trial findings	See Discussion section, 5th paragraph (‘Future research’), p.21
Interpretation	22	Interpretation consistent with results, balancing benefits and harms, and considering other relevant evidence	See Discussion section, 2nd paragraph (‘Effect evaluation and referrals’), p.17-18
**Other information**			
Registration	23	Registration number and name of trial registry	See end of Abstract, p.3
Protocol	24	Where the full trial protocol can be accessed, if available	See Methods section, 2nd paragraph (last section of ‘Study design’), p.7
Funding	25	Sources of funding and other support (such as supply of drugs), role of funders	See ‘Financial

*We strongly recommend reading this statement in conjunction with the CONSORT 2010 Explanation and Elaboration for important clarifications on all the items. If relevant, we also recommend reading CONSORT extensions for cluster randomised trials, non-inferiority and equivalence trials, non-pharmacological treatments, herbal interventions, and pragmatic trials. Additional extensions are forthcoming: for those and for up to date references relevant to this checklist, see www.consort-statement.org.
