# Supplementary material for: The Effectiveness of the BITSEA as a Tool to Early Detect Psychosocial Problems in Toddlers, a Cluster Randomized Trial
Source: PLoS One. 2015 Sep 18;10(9):e0136488. doi: 10.1371/journal.pone.0136488 (PMC4575038; doi:10.1371/journal.pone.0136488)
Supplement: S1 Protocol — (PDF) [file pone.0136488.s002.pdf]

Research Protocol

Validation of the Brief Infant-Toddler Social and Emotional Assessment (BITSEA) and evaluation of the BITSEA as screening tool in Youth Health Care for preschool children.

Dr. H. Raat
Februari 2009

Validation of the Brief Infant-Toddler Social and Emotional Assessment (BITSEA) and evaluation of the BITSEA as screening tool in Youth Health Care for preschool children.

December 2008

**Protocolnummer**	**Versie 1**
**Korte titel**	**BITSEA onderzoek**
**Datum**	**Februari 2009**
**Bestuurlijk verantwoordelijke**	**Prof dr. J.P. Mackenbach**<br>***Erasmus MC***<br>***Maatschappelijke gezondheidszorg***<br>***Postbus 2040***<br>***3000 CA Rotterdam***
**Projectleider**	***Dr. H. Raat***<br>***Erasmus MC***<br>***Maatschappelijke gezondheidszorg***<br>***Postbus 2040***<br>***3000 CA Rotterdam***<br>***T: 010-4088095***
**Mede project leider**	***Ir. W. Jansen***<br>***GGD Rotterdam-Rijnmond***<br>***Cluster jeugd, sectie M&R***<br>***Postbus 70032***<br>***3000 LP Rotterdam***<br>***T: 0104339968***<br>***E: jansenw@ggd.rotterdam.nl***
**Uitvoerend onderzoeker**	***Nog niet bekend***
**Project financier**	***ZonMW***<br>**Dossiernummer** 80-82435-98-8058

Table of contents

Summary……………………………………………………………………………3
Introduction………………………………………………………………………… 4
Objective (Research questions)………………………………………………………….. 5
Relevance……………………………………………………………………………6
Study design…………………………………………………………………………7
1. **Part 1** Test properties………………………………………………………………..9
1.1 Design and study population…………………………………………………9
1.2 Procedure………………………………………………………………….. 10
1.3 Measures…………………………………………………………………... 11
1.4 Data analysis………………………………………………………………. 11
1.5 Power considerations……………………………………………………… 12
1.6 Inclusion schedule and time schedule……………………………………...12
2. **Part 2** BITSEA as screening tool……………………………………………………13
2.1 Design and study population…………………………………………………13
2.2 Procedure………………………………………………………………….. 13
2.3 Measures…………………………………………………………………... 15
2.4 Data analysis………………………………………………………………. 15
2.5 Power considerations……………………………………………………… 15
2.6 Inclusion schedule and time schedule…………………………………….. 16
References…………………………………………………………………………...17
Publications………………………………………………………………………….18

ANNEX I Results of the systematic review…………………………………………... 21
ANNEX II Overview of study population and design………………………………….. 23
ANNEX III Inclusion schedule per month………………………………………………24
ANNEX IV Time Schedule……………………………………………………………....25

Summary
The prevalence of psychosocial problems among preschool children is already high. Early detection and treatment of emotional and behavioral problems have a positive effect on prognosis. However, currently many cases are being missed. The Invent-group strongly suggested to implement and to evaluate the test properties and impact of the Brief Infant-Toddler Social and Emotional Assessment (BITSEA) for the purpose of screening of emotional and behavioral problems among very young children (1-3 year old). The BITSEA can be applied by preventive Youth Health Care in the Netherlands (YHC; 'Jeugdgezondheidszorg 0-4 jarigen'; 'Consultatiebureaus'). YHC has a reach of over 90% of parents/children during the preschool years. We propose to validate and to evaluate the impact of the use of the BITSEA as screening tool by YHC at the routine visit of parent/children at age 24 months. The study will be done by 30 YHC-teams in the larger Rotterdam area, including both metropolitan areas with extensive and varied non-Dutch communities and non-metropolitan areas representative of the Dutch population at large. The 30 YHC-teams will include a total of net 3.000 parents/children in the study; there will be a follow-up of 12 months after the YHC-visit at age 24 months. At random 15 YHC-teams will be assigned to apply the BITSEA as a new screening tool at age 24 months (Intervention condition). The other 15 YHC-teams will apply 'usual care', i.e. application of the KIPPPI-'Consultatie Bureau Signalerings Instrument 2-jarige' (Control condition). In the Control group, parents will also complete the BITSEA (blinded for the YHC-professionals). The Child Behavior Checklist 1,5-5 (CBCL 1,5-5) (blinded for YHC) will be used to determine concurrent validity. Bilingual versions (Dutch; Turkish/ Arabic) will be offered. Additionally, a 'clinical' sample of (net) 100 circa 2-year olds (18-30 months old) referred to Youth Care and Youth Mental Health Services will be drawn through Youth Care/Mental Care agencies in the larger Rotterdam area. Parents of these children will also complete the BITSEA and KIPPPI-2, next to the CBCL 1,5-5. Data from the 'clinical' sample will be used to add to the validation of the screening tools. The study consists of two parts. In Part 1, test properties of the BITSEA will be determined in 2-year olds. In Part 2, the use of the BITSEA as screening tool in 2-year olds will be evaluated in comparison with the use of the KIPPPI-2. Data collection regarding the two parts of the study overlap. All 3.000 parents/toddlers in the study will complete the BITSEA and CBCL 1,5-5 at age 24 months, and at follow-up at age 36 months (in the Control group KIPPPI-2 will be used for screening at age 24 months); (medical) charts and registers of care will be reviewed at follow-up. Test properties of BITSEA will be described according to current standards (Part 1 of the study). Multi-level analyses will be applied in evaluating the impact of the BITSEA compared to the KIPPPI (Part 2 of the study).

Introduction

Early detection and treatment of emotional and behavioral problems in childhood may lead to considerable benefits regarding development, well-being and health (Durlak et al, 1998; Elliot et al, 2002). Given the fact that both internalizing and externalizing psychosocial problems already have a high prevalence among preschool children, is has been hypothesized that early detection and interventions should start at young age (Skovgaard et al, 2004; Carter et al, 2004; Zeijl et al, 2005). Currently however, many cases of psychosocial problems in (very) young children are being missed in the Netherlands (Reijneveld et al, 2004).

The Dutch Invent-group recently stated that collaborative efforts are needed to implement and evaluate promising ('veelbelovend'), uniform strategies by Youth Health Care (YHC; 'Jeugdgezondheidszorg') and other involved organizations to detect high-risk child-rearing situations and act upon it in a coherent, effective way (Hermanns et al, 2005). The Invent-group thoroughly assessed existing instruments to systematically screen (young) children for the presence of emotional and behavioral problems. The Invent-group identified the Brief Infant-Toddler Social and Emotional Assessment (BITSEA) as a very promising instrument to detect psychosocial problems among the youngest children, those aged 1-3 years old (Hermanns et al, 2005; page 39).

The BITSEA, a 42-item questionnaire for parents with multiple-choice answer options, is intended for early detection and screening of externalizing and internalizing problems of children aged 12 month to 3 years (Carter et al, 2004; Briggs-Gowan et al, 2004). The BITSEA scoring system provides a Problem Total Score and a Competence Total Score. A Dutch translation of the original English version is already available (Hermanns et al, 2005); Turkish and Arabic versions will be provided in this project to be applied in multi-ethnic populations The Invent-group considers the BITSEA a potential powerful and proper screening tool for the youngest children (1-3 years), preceding the SDQ (Strengths and Difficulties Questionnaire) that is (potentially) appropriate for children at least 3 years old. The BITSEA showed, in other countries, a good sensitivity, specificity, predictive value and other satisfying psychometric properties (Briggs-Gowan et al, 2004; Hermanns et al, 2005, page 110-111). The full-length version, the ITSEA, has been applied as an epidemiological measure (not as a screening tool) in the SCP-TNO study 'Kinderen in Nederland' ('Children in the Netherlands') (Zeijl et al, 2005). Please refer to the systematic review regarding the ITSEA and BITSEA that is attached to this proposal ('Beknopte systematische zoekactie').

Despite its potential, the BITSEA has not yet been implemented and evaluated as screening tool in the setting of regular preventive Youth Health Care for preschool children that is offered to all families and has a reach of over 90%. Therefore, the Invent-group has strongly proposed to further develop and test the BITSEA in the Dutch setting and to evaluate both test properties and its potential for implementation in daily practice of Dutch Youth (Health) Care (Hermanns et al, 2005; page 39). We propose to do so in this study in the setting of YHC for preschool children ('Consultatiebureaus') in the larger Rotterdam area, including both metropolitan areas with extensive and varied non-Dutch communities and non-metropolitan areas representative of the Dutch population at large.

Dutch YHC provides an efficient framework for early detection and management of psychosocial problems in young children (Update ZonMw Programmeringsstudie, 2005). Several prior studies have shown that it is highly feasible to implement parent-completed screening tools such as the BITSEA in YHC for preschool children (Reijneveld et al, 2004; Zeijl et al, 2005; Wiefferink et al, 2006). YHC is in the process of becoming an integral part

of the whole chain-of-care for children and families. In the larger Rotterdam area, the setting of this study, the first ‘Centers for Youth and Families’ have already started. Furthermore, in the larger Rotterdam area, an extremely helpful regional Youth Care Registry has been implemented, promoting collaboration and quick referral between YHC and other relevant agencies (SISA/SJS registries). Also, in this area, already protocols have been implemented for the management of psychosocial problems and referral to evidence-based interventions (SJS, 2006).

In this study we will validate the BITSEA in the setting of YHC in a large varied population (part 1 of the study) and we will evaluate the impact of the use of the BITSEA as screening tool by YHC at age 2 years; the impact of the BITSEA will be compared with ‘usual care’, which in the Rotterdam area is application of the KIPPPI-‘Consultatie Bureau Signalerings Instrument 2-jarige’ (see ‘Strategy’) (part 2 of the study).

Objective

The objective of this study is to validate the Brief Infant-Toddler Social and Emotional Assessment (BITSEA) (Part 1 of the study) and to evaluate the impact of the use of the BITSEA as screening tool by preventive Youth Health Care (YHC; ‘Jeugdgezondheidszorg’) at age 24 months, compared to ‘usual care’, i.e. application of the KIPPPI-‘Consultatie Bureau Signalerings Instrument 2-jarige’ (Part 2 of the study). In the study, the Child Behavior Checklist 1,5-5 (CBCL 1,5-5) will be used to determine concurrent validity of the BITSEA. Furthermore, in the study, data of a ‘clinical’ sample will be used to add to the validation of the BITSEA. In Part 2 of the study follow-up measures at age 36 months will be used.

<u>Research questions:</u>

PART 1 OF THE STUDY: ‘TEST PROPERTIES’

1. What are the sensitivity, specificity, predictive value of test results, and other psychometric test properties (including reliability, factorial validity, concurrent and discriminative validity) of the BITSEA?

PART 2 OF THE STUDY: ‘BITSEA AS SCREENING TOOL’

2. What is the outcome at 12-months follow-up of the use of the BITSEA in YHC centers that apply it compared to YHC centers that apply the KIPPPI-‘Consultatie Bureau Signalerings Instrument 2-jarige’ (‘usual care’) in terms of (a) emotional and behavioral problems at follow-up, and (b) delivery of care and interventions (number, type, adherence, rapidity)?

3. What is the feasibility of the use of the BITSEA as screening tool according to professionals and parents?

4. What are the costs of the use of the BITSEA per detected case and what is the ratio between costs and effects of BITSEA use in terms of improvement of indicators of psychosocial health at follow-up compared to usual care?

We will explore test properties, outcomes and process characteristics for subgroups non-Dutch children.

Relevance

This study concerns the validation and evaluation of a prioritized early detection instrument that will be completed by parents of preschool children, discussed with the Youth Health Care professionals (generally specialized Youth Health Care nurses and specially trained physicians), enabling shared decisions with parents concerning the well being of their child and family. This proposal therefore connects to the main aim of the ZonMW program Care for Youth.

This proposal specifically fits into Priority [A] of the recently established ZonMw program Care for Youth (Zorg voor Jeugd); i.e. 'Drive back (risks for) parenting problems regarding children below age 7 years'. Screening for emotional and behavioral problems by preventive Youth Health Care at child age 24 months using the BITSEA, followed by timely referral and adequate follow-up, may effectively prevent risk full parenting situations, and may effectively improve optimal development of young children.

The BITSEA is recommended by the Dutch Invent-group, but there is little knowledge about the psychometric characteristics in the setting of day-to-day, regular preventive Youth Health Care in the Netherlands. Worldwide there are only very few longitudinal studies with the BITSEA, which are necessary to evaluate the predictive validity of this promising instrument. In this study we will evaluate the impact of screening using the BITSEA at child age 2 years in routine preventive Youth Health Care with a 12 months follow-up, compared to screening using the KIPPPI-2, which constitutes 'usual care'. This knowledge will be important for decisions by RIVM Center for Youth Health and other responsible agencies about nation-wide implementation of screening for emotional and behavioral problems using the BITSEA at preschool age in preventive Youth Health Care centers, and in the near future centers for Youth and Family.

The study group consists of toddlers 2-3 years old, with a Dutch as well as a non-Dutch background in the varied region of Rotterdam Rijnmond (inner city areas as well as rural areas are represented). The young age of our study population (age 2-3 years), and the high proportion of non-Dutch children in the study (estimated to constitute 25% of the whole study population), connects well to two other priorities in the ZonMw program Care for Youth.

Note: In the city of Rotterdam, about 50% of the 2 year olds are of non-Dutch origin; i.e. circa 10% of Surinam origin, 15% of Turkish origin, 10% of Moroccan origin and 15% of other ethnic minority groups.

Using the BITSEA as a screening tool in the preventive Youth Health Care for preschool children has important implications for the partners in the so called chain-of-care for children and families. Youth Care is a very important partner in this chain, and for this reason plays an important role in future Centers for Youth and Family. The screening tools used by preventive Youth Health Care need to be feasible and proper instruments for the entire chain-of-care. This study will pay attention to these prerequisites.

Study Design

The study consists of two parts. In Part 1, test properties of the BITSEA will be determined in 2-year olds (Research question 1). In Part 2 the use of the BITSEA as screening tool in 2-year olds will be evaluated in comparison with the use of the KIPPPI-'Consultatie Bureau Signalerings Instrument 2-jarige', which constitutes 'usual care' (Research questions 2-4).

The study will take place in the (broad) Rotterdam Rijnmond area, including the Dordrecht area, Capelle a/d IJssel, the Rotterdam area, and the Schiedam/Vlaardingen area.
The GGD Public Health Service Rotterdam Rijnmond participates in the study. The GGD organizes the Youth Health Monitor, which will contain the screening tools for emotional and behavioral problems that parents complete, and bring along to their regular visit to the preventive Youth Health Care (YHC) center when their child is 24 months old. The four practice organizations for preventive Youth Health Care for preschool children in the study area (Ouder- en Kindzorg; Consultatiebureaus) will participate in the study; their professionals (specialized YHC-nurses, in cooperation with specifically trained YHC-physicians) use the screening tools for emotional and behavioral problems, and decide on referral and follow-up care.

In the study, the Child Behavior Checklist 1,5-5 (CBCL 1,5-5) will be used to determine concurrent validity of the BITSEA. Additionally, in the study data of a 'clinical' sample will be used to add to the validation of the BITSEA. In Part 2 of the study follow-up measures at age 36 months will be used, including repeated measurements of BITSEA and CBCL 1,5-5 and (medical) chart review and review of a comprehensive registers of care in the Rotterdam Rijnmond area.

Data collection regarding Part 1 and Part 2 of the study overlap.
Please refer to the attached Annex with a schematic overview of study populations and design of Part 1 and Part 2 of the study. Please also refer to the attached Annex with a systematic review of the literature on the BITSEA.

Theoretical model applied in the study:
The theoretical model applied in this study, is the model of screening for abnormalities, followed by referral, diagnosis and early treatment if indicated (see Mackenbach et al, 2008, page 240-253). This model of screening has been developed in the medical setting, e.g. for early detection and treatment of tuberculosis (mid 20th century) or early detection and treatment of specific cancers (late 20th century). Late 20th century, early 21st century this model was transferred to the early detection, referral, diagnosis and treatment of emotional and behavioral problems (and even risks for the development of such problems) in childhood (Hermanns et al, 2005).

The underlying assumption of the model of screening is that early detection, diagnosis and treatment (i.e. earlier than would have happened in the 'natural course of events', i.e. without the screening procedure) results in outcomes that are better compared to outcomes in the case of no screening. However, the model of screening acknowledges that depending on the condition for which is being screened, the screening procedure and the setting, a screening procedure may be ineffective or may even be harmful. This is reflected in a quote by Sir Muir Gray 'all screening programs do harm; some do good as well' (cited by Mackenbach et al, 2008, page 242). The circumstances that are related to the (potential) outcomes of a screening are summarized in 10 criteria for effective screening that were proposed by Wilson and Jungner (cited by Mackenbach et al, 2008, page 250):

1. The problem/disease should present a public health problem (i.e. a relevant problem at societal level).
2. An acceptable and effective treatment should be available.
3. Ample facilities for further diagnosis and treatment should be available.
4. The problem/disease should have a clear 'latent' phase (i.e. a - hypothetical, ideal - screening test should be helpful to discover a problem/disease that otherwise would have remained unseen/undiagnosed).
5. The test properties (among which sensitivity, specificity, predictive value of test results) should be adequate.
6. The screening test should be acceptable for the population.
7. The natural course of the problem/disease should be known (i.e. the course of the problem/disease in the absence of screening).
8. Protocols regarding criteria for referral and further diagnosis, and criteria for diagnosis and treatment should be available.
9. The costs of the whole screening procedure (including referral, diagnosis, and treatment) should be acceptable in the whole context of (health) care.
10. The screening process should be well-organized and should be embedded in a continuous program (i.e. not an ad-hoc procedure).

Part 1 and Part 2 of this study will clearly contribute to the empirical knowledge needed to decide on future implementation of screening for emotional and behavioral problems in 2-year olds by using the BITSEA in preventive Youth Health Care in the Netherlands, given the 10 criteria of Wilson and Jungner.

Adequate test properties of the BITSEA (criterion 5) have been established in the U.S.. This study will contribute to the knowledge needed to decide on the plausibility of the BITSEA screening in the Netherlands by evaluating the test properties of the BITSEA in the context of day-to-day practice of preventive Youth Health Care in the Netherlands.

Hypotheses applied in Part 2 of the study:

We hypothesize that use of the BITSEA as screening tool compared to KIPPPI-2, results in more appropriate and timely care; and that fewer problems indicated by the CBCL 1,5-5 will be present at 1 year follow-up in the BITSEA group (Intervention condition) compared to the KIPPPI-2 group (Control condition).

We also hypothesize the (relative) costs of the use of the BITSEA to be lower than the costs of the KIPPPI-2 as screening tool.

PART 1 OF THE STUDY: ‘TEST PROPERTIES’

In the Part 1 of the study test properties, including psychometric characteristics, sensitivity, specificity and predictive value of the BITSEA will be studied (Research question 1).

Please refer to the attached Annex with a schematic overview of study populations and design of Part 1 and Part 2 of the study. Please also refer to the attached Annex with a systematic review of the literature on the BITSEA, including a description of the instrument. (see Proposal zonmw)

1.1 Design and study population (see annex with overview)

A. Community sample including parents that visit regular preventive YHC at age 2 years

A large community sample of 2-year olds (and their parents) in the Rotterdam Rijnmond region with both metropolitan areas with extensive and varied non-Dutch communities and non-metropolitan areas representative of the Dutch population at large, will be used in Part 1 of the study. The study will be carried out by 30 YHC-teams from 4 organizations for preventive YHV in the region. They will obtain informed consent from parents with 2-year olds eligible for the routine health assessment by preventive YHC at age 24 months.

Net 3.000 parents/2-year olds will complete the BITSEA in the course of the study at age 24 months.

Please note that for the purpose of Part 2 of the study (see below) from the total of 30 participating YHC-teams in the study, at random 15 YHC-teams will be assigned to apply the BITSEA as a new screening tool at age 24 months (Intervention condition). The other 15 YHC-teams will apply 'usual care', i.e. application of the KIPPPI-'Consultatie Bureau Signalerings Instrument 2-jarige' (Control condition).

In the Control group in which the KIPPPI-2 is used as a screening tool, the parents will also complete the BITSEA (blinded for the YHC-professionals; in the Control group the YHC-professionals will not use the BITSEA results in the process of health assessment and early detection of emotional and behavioral problems).

In addition to and separately from the Youth Health Monitor, the 3.000 participating parents of 2-year olds in the study will be invited to complete the Child Behavior Checklist 1,5-5 (CBCL 1,5-5), a parent-completed 99-item questionnaire. The CBCL 1,5-5 will be applied to determine the concurrent validity of the BITSEA. The CBCL 1,5-5 is an internationally validated measure that allows making a (provisional) diagnosis respectively problem definition; the CBCL is widely used as gold standard in validation studies (Carter et al, 2004; Achenbach et al, 2000; Reijneveld et al, 2004; Zeijl et al, 2005).

Furthermore, during the regular visit at the YHC center at age 2 years, the YHC-professional will register (routinely) whether emotional or behavioral problems are present, and whether follow-up care or referral is recommended, according to professional judgment based on the protocols (in the Intervention group the BITSEA will be used; in the Control group the KIPPPI-2). Bilingual versions (Dutch; Turkish/Arabic) of the questionnaires will be offered.

B. ‘Clinical’ sample

Additionally, a ‘clinical’ sample of net 100 toddlers that are circa 2 years old (18-30 months old) and that have been referred to Youth Care or Youth Mental Health Services will be drawn through Youth Care/Mental Care agencies in the Rotterdam Rijnmond area. Parents of these 2-year olds will also complete the BITSEA, the KIPPPI-2, and the CBCL 1,5-5. Given informed consent by the parents, the diagnosis regarding the child will be retrieved from the (medical) chart. Data from the ‘clinical’ sample will be used to add to the validation of the screening tools.

1.2 Procedure

In the Rotterdam Rijnmond area 30 YHC-teams will participate in the study. The YHC-teams will be selected from four practice organizations for preventive Youth Health Care for preschool children (Ouder- en Kindzorg organisaties; Consultatiebureaus). These organizations are Ouder- en Kindzorg Rotterdam (OEK) with circa 28 YHC-teams and 6.500 births per year; De Stromen Opmaat Groep in Dordrecht area with circa 14 YHC-teams and 3.400 births per year; Careyn in Schiedam/Vlaardingen area with circa 7 YHC-teams and 2.000 births per year; and Thuiszorg de Zellingen in Capelle a/d IJssel with circa 3 YHC-teams and 700 births per year.

The Youth health Care professionals will inform parents of 2-year olds about the study, prior to their regularly scheduled routine visit at age 24 months. The parents will be invited to provide written informed consent.

As stated above, for the purpose of Part 2 of the study (see below), at random 15 teams will be assigned to apply the BITSEA as screening tool using the so-called Youth Health Monitor (YHM) at age 24 months. Fifteen other YHC-teams (randomized) will not use BITSEA as screening tool, but the parents in the Control group will be invited to additionally complete the BITSEA, but its results will not be used by the YHC-professional (blinded).

According to regular practice parents that visit the YHC-center when the child is 24 months old, complete the Youth Health Monitor (a health questionnaire); for Turkish and Moroccan parents bilingual forms are available; if necessary parents get assistance at the YHC-center.

Separately from the Youth Health Monitor, at child age 24 months all participating parents will be invited to complete a concise additional questionnaire anonymously. In the Intervention group this questionnaire contains the CBCL 1,5-5. In the Control group this questionnaire contains the CBCL 1,5-5 and the BITSEA. As stated above, scores from this additional questionnaire at age 24 months will not be used in the routine health assessment (see schematic Overview of the study in Annex).

Numbers in the study:

A total of 6.000 parents/toddlers will be invited by the 30 participating Youth Health Care teams in the study. Assuming (at least) informed consent by 50%, 3.000 parents/toddlers will be included. All participating YHC-teams and each participating YHC-professional in the study will receive handy, clear, written instructions (fact sheets/charts) about the role of each participant in the study. Instructions will be discussed during regular professional meetings (prior to the start of the inclusion, and also repeatedly during the study). Specific competences that are necessary will be trained (but this is especially relevant for Part 2 of the study; see paragraph 2.2).

Independent of this instruction/training with regard to the study, in a separate, independent proposal, we have presented a plan for activities to promote the readiness for doing research and to promote the willingness to participate in an optimal way in effectiveness studies by YHC-professionals that will cooperate in the study. For that separate purpose we applied for a so-called “Praktijkbonus” (additional financial support) that will be used to support these efforts of the four practice organizations that cooperate in the study (please refer to the Annex ‘Praktijkbonus’ attached to this proposal).

1.3 Measures at age 24 months
(community sample ~ n=3.000; and ‘clinical’ sample ~ n=100)

Note: These measures are only available providing informed consent by the parents

- BITSEA
- KIPPPI-2 (only in the Control group, and in the ‘clinical’ sample; not in the Intervention group)
- CBCL 1,5-5
- Parent report of use of (mental) health services and other professional support
- Diagnosis and decisions regarding follow-up care or referral by the YHC-professional at regular YHC-visit at age 24 months (Intervention group and Control group; if available in ‘clinical’ sample) (Reijneveld et al, 2004; Zeijl et al, 2005)
- Entries in the child’s medical record/Youth Care record (‘clinical’ sample only)
- Entries in the child’s regional Youth Care Registries (SISA/SJS) (SJS, 2006)
- Socio-demographic characteristics

1.4 Data analysis

Test properties of the BITSEA will be described, including internal consistency, inter-rater reliability, factorial validity, concurrent validity with the CBCL 1,5-5, and discriminative validity (BITSEA results compared to professional findings). Receiver Operating Characteristic (ROC) curves for all possible cut-off points will be made available by using results of the ‘clinical’ sample. See Mackenbach et al (2008), page 240-253 for description of the test properties and procedures to evaluate the BITSEA as screening tool.

Two criteria will be used in this analysis: (a) a ‘clinical score’ on the CBCL 1,5-5; and (b) referral to Youth Care/Child Mental Health Care (and additionally other professional diagnoses by YHC-professionals or Youth Care/Mental Health Care professionals.

Normative data and cut-offs will be produced will be produced based on the total community sample. All test results will be explored and described for subgroups of children from ethnic minorities.

1.5 Power considerations

Since in Part 1 of the study no hypotheses are tested, power calculations are not really applicable. Nevertheless remarks can be made on the precision of the estimates this part of the study will yield.

A sample of 3.100 toddlers (3.000 of the community sample and 100 of the clinical sample) will allow to estimate the area under the Receiver Operating Characteristic (ROC) curve of about 0.90 with a 95% confidence interval of +/- 0.02 (Hanley et al, 1982). A sub-sample size of 217 toddlers (e.g. a specific ethnic sub-group of 7% of the whole population, assuming equal prevalence of emotional and behavioral problems) will allow to estimate the area under the ROC curve of about 0.90 with a 95% confidence interval of +/- 0.08.

For evaluating concurrent validity of the BITSEA, correlations between specific BITSEA scores and specific, hypothesized CBCL 1,5-5 scores will be computed. Even in a sub-sample of 500 toddlers we can estimate a correlation of 0.20 (or higher) with a power of 99% (alpha = 0.05; one sided testing). An even smaller sub-sample of 50 toddlers (of a small specific ethnic sub-group for instance) will allow to estimate a correlation of 0.40 with a power of 90% (alpha=0.05; one sided testing).

Based on the entire community sample (n=3.000) the p90 of the BITSEA Problem Total Score of 2-year olds can be estimated with a precision of +/- 3% for boys and girls separately.

1.6 Inclusion schedule and time schedule of the study

An Inclusion schedule by month and a time schedule by month of Part 1 of the study are included as Annex to the protocol.

PART 2 OF THE STUDY: ‘BITSEA AS SCREENING TOOL’

Part 2 of the study is intended to answer Research questions 2, 3, and 4 (See paragraph on Objectives). Part 2 will establish the outcomes after a follow-up of 12 months, and the feasibility and costs of the BITSEA screening tool. The BITSEA will be applied by 15 YHC centers in the study, and the results will be compared with the results of ‘usual care’ in 15 other YHC centers (based on randomization) in the varied Rotterdam Rijnmond region. ‘Usual care’ implies the application of the KIPPPI-2.

Please refer to the attached Annex with a schematic overview of study populations and design regarding Part 2 of the study. Please also refer to the attached Annex with a systematic review of the literature on the BITSEA, including results of follow-up studies, and the absence of such studies in the Netherlands.

2.1 Design and study population

The study is designed as a cluster-randomized controlled trial (cRCT), with a baseline and a follow-up measurement after 12 months, and a Control condition (screening using the KIPPPI-2) and an Intervention condition (screening using the BITSEA). The YHC team is the unit of randomization (Campbell et al, 2004). The four collaborating preventive YHC organizations for preschool children in the Rotterdam Rijnmond region will participate with 30 YHC-teams. So, 15 YHC-teams will belong to the Intervention group (BITSEA screening) and 15 YHC-teams will belong to the Control group (KIPPPI-2 screening); based on randomization of the teams.

2.2 Procedure

Prior to the regularly scheduled, routine visit to the preventive YHC-center at child age 24 months, all parents of a 2-year old will be fully informed and invited to participate with their child in the study; written informed consent will be obtained.

As described in paragraph 1.2 (above) prior to the visit, the parents complete the Youth Health Monitor questionnaire as part of the normal routine at the YHC-centers. In the Control group, as usual, the Youth Health Monitor questionnaire includes the KIPPPI-2. In the Intervention group the Youth Health Monitor includes a new questionnaire, i.e. the BITSEA.

As stated above bilingual versions (Dutch; Turkish/Arabic) are available and help can be provided by the YHC-center as part of the regular procedures.

Separately from the Youth Health Monitor, at child age 24 months all participating parents will be invited to complete a concise additional questionnaire anonymously. In the Intervention group this questionnaire contains the CBCL 1,5-5. In the Control group this questionnaire contains the CBCL 1,5-5 and the BITSEA. As stated above, scores from this additional questionnaire at age 24 months will not be used in the routine health assessment (see schematic Overview of the study in Annex).

Trained YHC-professionals (see below for details regarding the training) use either the BITSEA or the KIPPPI-2 as screening tool during the visit. According to the existing protocols, professionals will make use of cut-off points based on the 90th percentile (p90) of age-appropriate normative samples for interpretation of test results, next to other professional

observations in the Intervention group (BITSEA; see Briggs-Gowan MJ et al. Brief Infant Toddler Social and Emotional Assessment - BITSEA Manual. New Haven: Yale University; 2005) as well as in the Control group (KIPPPI-2; see the website of KIPPPI-2 http://www.kipppi.nl/; Jeugdmonitor Rotterdam, 2004).

Twelve months after the 24 months YHC-visit, so at age 36 months, all participating parents in the study will be invited to complete a follow-up questionnaire including BITSEA, CBCL 1,5-5, and items on use of care, items on satisfaction with YHC, and if applicable referral and follow-up care, and satisfaction regarding referral and follow-up.

Furthermore, from the files and registry forms the diagnosis and decisions regarding follow-up care or referral by the YHC-professional at the regular YHC-visit at age 24 months will be retrieved, and at age 36 months, the entries in the child's preventive Youth Health Care record (Electronic Medical File; 'Elektronisch Kinddossier') will be retrieved, as well entries in the child's region-wide registry of Youth Care (providing informed consent by the parents).

Numbers in the study:

A total of 6.000 parents/toddlers will be invited. Assuming (at least) informed consent by 50%, 3.000 parents/toddlers will be included. Allowing dropout between baseline and follow-up of 30%, we expect complete data regarding 2.100 parents/toddlers after follow-up.

As stated in paragraph 1.2, all participating YHC-teams and each participating YHC-professional in the study will receive handy, clear, written instructions (fact sheets/charts) about the role of each participant in the study. Instructions will be discussed during regular professional meetings (prior to the start of the inclusion, and also repeatedly during the study). The competences that are necessary to use the BITSEA as a screening tool will be trained in the Intervention group. All YHC-professionals in the Intervention group (mostly the specialized YHC-nurses) will be involved in this training. Since the participating YHC practice organizations contemplate implementing the BITSEA regardless of this study, this training will be scheduled in the normally available time for professional training and education in the organization.

As stated in paragraph 1.2, independent of this instruction/training with regard to the study, in a separate, independent proposal, we have presented a plan for activities to promote the readiness for doing research and to promote the willingness to participate in an optimal way in effectiveness studies by YHC-professionals that will cooperate in the study. For that separate purpose we applied for a so-called "Praktijkbonus" (additional financial support) that will be used to support these efforts of the four practice organizations that cooperate in the study (please refer to the Annex 'Praktijkbonus' attached to this proposal).

Note regarding the KIPPPI-2:

The KIPPPI-'Consultatie Bureau Signalerings Instrument 2-jarige' will be applied in the 'usual care' group. It is an alternative screening tool for 2-year olds developed by Dr. P. Kousemakers (see http://www.kipppi.nl/), has been applied regularly in YHC until now, but was NOT advised by the Invent-group given lack of (international) evidence for validity and effectiveness (Hermanns et al, 2005; Jeugdmonitor Rotterdam, 2004).

2.3 Measures

Note: These measures are only available providing informed consent by the parents

Outcomes

- CBCL 1,5-5 at baseline and 12-month follow-up (CBCL 1,5-5 scores are considered the primary outcomes)
- BITSEA at baseline and 12-month follow-up
- YHC-professional problem diagnosis and treatment/referral decisions at baseline and during follow-up (Reijneveld et al, 2004; Zeijl et al, 2005)
- Entries in the regional Youth Care Registries (SISA/SJS) at baseline and during follow-up (SJS, 2006)
- Child health-related quality of life (General health perceptions subscale of the ITQOL; Raat et al, 2006)

Co-variables (at baseline and at follow-up)

- Course of pregnancy, medical and family history from medical file; Socio-demographic and environmental variables (Reijneveld et al, 2004; Zeijl et al, 2005)

Process measures

- Adherence to the distinct elements of the early detection
- Appreciation/satisfaction regarding the early detection
- Time investment/other costs of the procedure.

2.4 Data analysis

Multilevel analyses will be applied to allow for dependency between the individual measurements within the randomized YHC-teams (Campbell et al, 2004; Twisk, 2006). Multilevel linear regression analyses will be conducted for the continuous outcome variables with group (intervention or control) as independent variable and baseline values and potential confounders as covariates. Multilevel logistic regression will be performed in case of dichotomous outcome variables. Interaction effects by gender and ethnic background will be explored by introducing an interaction term into the regression analyses (Twisk, 2006).

Adherence to and satisfaction with the distinct elements of the early detection procedure by parents and professionals will be described.

Costs and effects will be evaluated for the purpose of a cost-benefit analysis.

2.5 Power considerations

At follow-up, we expect complete data of 2.100 parents/toddlers, equally divided over Intervention/Control group. We take alpha of 0.05 and power of 0.8. We apply a correction factor to account for the cluster design (assuming Intra Class Correlation Coefficient to be 0.10). Within the whole study population (n=2.100 for Intervention and Control group combined), for the CBCL 1.5-5 total problems score the study can detect at follow-up a minimal difference between intervention/control group of 8.0 points (SD=26.5) (Achenbach et al, 2000).

Within the sub-group in the study with psychosocial problems at baseline (assumed to be 10% of the population at baseline; so 210 in Intervention and Control group combined), for the CBCL 1.5-5 total problems score, the study can detect at follow-up a minimal difference between intervention/control group of 11.9 points (SD=26.5) (Achenbach et al, 2000).

2.6 Inclusion schedule and time schedule of the study

A time schedule by month of Part 2 of the study is included as Annex to the protocol.
An Inclusion schedule by month of the study is also included as a separate Annex to the protocol.

REFERENCES (AUTHOR, YEAR)

Achenbach TM, Rescorla LA, *Manual for the aseba preschool forms & profiles.* University of Vermont, Research Center for Children, Youth, & families: Burlington, VT; 2000.

Basistakenpakket Jeugdgezondheidszorg 0-19 jaar. Ministerie van Volksgezondheid Welzijn en Sport; 2002.

Briggs-Gowan MJ et al, *The Brief Infant-Toddler Social and Emotional Assessment: screening for social-emotional problems and delays in competence.* J Pediatr Psychol, 2004. **29**(2): p. 143-55.

Campbell MK et al, CONSORT statement: extension to cluster randomised trials. BMJ 2004, 328(7441):702-708.

Carter AS et al, *Assessment of young children's social-emotional development and psychopathology: recent advances and recommendations for practice.* J Child Psychol Psychiatry, 2004. **45**(1): p. 109-34.

Durlak JA et al, *Evaluation of indicated preventive intervention (secondary prevention) mental health programs for children and adolescents.* Am J Community Psychol, 1998. **26**(5): p. 775-802.

Elliot J et al, *Evaluation of a community intervention programme for preschool behaviour problems.* J Paediatr Child Health, 2002. **38**(1): p. 41-50.

Hanley, J.A. and B.J. McNeil, The meaning and use of the area under a receiver operating characteristic (ROC) curve. Radiology, 1982. 143(1): p. 29-36.

Hermanns J, Ory F, Schrijvers G, *Helpen bij opgroeien en opvoeden: eerder, sneller, beter. Een advies over Vroegtijdige signalering en interventies bij opvoed- en opgroeiproblemen. Advies uitgebracht op verzoek van Staatssecretaris drs. C. Ross-van Dorp.* Julius Centrum: Utrecht; 2005.

Jeugdmonitor Rotterdam, Onderzoek peuters. GGD, 2004.

Mackenbach, J.P., P.J. van der Maas (red.), Volksgezondheid en gezondheidszorg. Elsevier, Maarssen, 2008.

Operatie Jong. Informatie over operatie Jong. Rotterdam, 2006.

Raat H et al, *Reliability and validity of the Infant and Toddler Quality of Life Questionnaire (ITQOL) in a general population and respiratory disease sample.* Qual Life Res, 2006.

Reijneveld SA et al, *Identification and management of psychosocial problems among toddlers in Dutch preventive child health care.* Arch Pediatr Adolesc Med, 2004. **158**(8): p. 811-7.

Skovgaard AM et al, *Assessment and classification of psychopathology in epidemiological research of children 0-3 years of age: a review of the literature.* Eur Child Adolesc Psychiatry, 2004. **13**(6): p. 337-46.

Snel / Safe Jeugd Signaal. Wanneer en hoe melden in SJS. Versie 1.0. 2006, JONG Advies voor Jeugd en Ouders: Rotterdam.

Twisk JWR: Applied Multilevel Analysis. University Press Cambridge; 2006.

Update ZonMw Programmeringsstudie Effectiviteitsonderzoek Jeugdgezondheidszorg. Neppelenbroek SE, Wijngaarden JCM van, Lim-Feijen JF, Leerdam FJM van, Raat H, Hirasing RA. GGD Nederland, LVT, VU medisch centrum, Erasmus MC, TNO-KvL; 2005.

Wiefferink CH et al, *Screening for psychosocial problems in 5-6-year olds: a randomised controlled trial of routine health assessments.* Patient Educ Couns, 2006. **60**(1): p. 57-65.

Zeijl E et al, *Kinderen in Nederland (Children in the Netherlands).* SCP/TNO Kwaliteit van Leven: Den Haag/Leiden; 2005.

Publications

Neppelenbroek, S.E., van Wijngaarden JCM, Lim-Feijen JF, van Leerdam F.J.M., Raat H, HiraSing R.A., Update Programmeringsstudie effectonderzoek jeugdgezondheidszorg 0-19 jaar. Utrecht: GGD Nederland; 2005.

Mackenbach JP, Kunst AE, Cavelaars AEJM, Groenhof F, Geurts JJM and the EU Working Group on Socioeconomic Inequalties in Health. Socioeconomic inequalities in morbidity and mortality in Western Europe. Lancet 1997;349:1655-1659

C.M.P. Buysse, H. Raat, J.A. Hazelzet, W.C.J. Hop, M. Maliepaard, K.F.M. Joosten, Surviving meningococcal septic shock: health consequences and quality of life in children and their parents up to 2 years after PICU discharge. Crit Care Med, 2008 Feb;36(2):596-602.

C.M.P. Buysse, H. Raat, J.A. Hazelzet, L.C.A.C. Vermunt, E.M.W.J. Utens, W.C.J. Hop, K.F.M. Joosten, Long-term health-related quality of life in survivors of meningococcal septic shock in childhood and their parents. Qual Life Res, Dec 2007, 16(10):1567-76.

E.W.M. Troe, H. Raat, V.W.V. Jaddoe, A. Hofman, C.W.N. Looman, H.A. Moll, E.A.P. Steegers, F.C. Verhulst, J.C.M. Witteman, J.P. Mackenbach, Explaining differences in birth weight between ethnic populations. The Generation R Study. BJOG, December 2007, 114(12)1557-65.

E.W.M. Troe, H. Raat, V.W.V. Jaddoe, A. Hofman, E.A.P. Steegers, F. C. Verhulst, J.C.M. Witteman, J.P. Mackenbach, Smoking during pregnancy in ethnic populations. The Generation R study. Nicotine & Tobacco Research, 2007. In press.

I. Vogel, J. Brug, E.J. Hosli, C.P.B. van der Ploeg, H. Raat, MP3 players and hearing loss: adolescents' perceptions of loud music and hearing conservation. The Journal of Pediatrics, 2007. In press. (DOI: 10.1016/j.jpeds.2007.07.008)
A.D. Mohangoo, H.J. de Koning, R.T. Mangunkusumo, H. Raat, Health-related quality of life in adolescents with wheezing attacks. J Adolesc Health, November 2007: 41(5): 464-71.

R. Oostenbrink, K. Spong, A. De Goede - Bolder, J.M. Landgraf, H. Raat, H.A. Moll, Parental reports of health-related quality of life in young children with neurofibromatosis type 1: influence of condition specific determinants. J Pediatr, August 2007. 151(2): p. 182-6, 6 e1-2.

I. Vogel, J. Brug, C.P. van der Ploeg, H. Raat, Young People's Exposure to Loud Music A Summary of the Literature. Am J Prev Med, Aug 2007. 33(2): p. 124-33.

A. M. Abdalla, A. F. Al-Kaabba, A. A. Saeed, B. M. Abdulrahman, H. Raat, Gender differences in smoking behavior among adolescents in Saudi Arabia. Saudi Med J, Jul 2007. 28(7): p. 1102-8.

Beirens, T. M., Brug, J., van Beeck, E. F., Dekker, R., den Hertog, P., Raat, H., Assessing psychosocial correlates of parental safety behaviour using Protection Motivation Theory: stair gate presence and use among parents of toddlers. Health Educ Res, Oct 18, 2007, 0268-1153 (Print).

T.M.J. Beirens, J. Brug, E.F. van Beeck, R. Dekker, R.E. Juttmann, H. Raat, Presence and use of stair gates in homes with toddlers (11-18 months old). Accid Anal Prev, Sep 2007, 39(5):964-8.

H. Raat, R.T. Mangunkusumo, A.D. Mohangoo, E.F. Juniper, J. Van Der Lei, Internet and written respiratory questionnaires yield equivalent results for adolescents. Pediatr Pulmonol, Apr 2007. 42(4): p. 357-61.

H. Raat, R.T. Mangunkusumo, J.M. Landgraf, G. Kloek, J. Brug, Feasibility, reliability, and validity of adolescent health status measurement by the Child Health Questionnaire Child Form (CHQ-CF): internet administration compared with the standard paper version. Qual Life Res, May 2007. 16(4): p. 675-85.

Raat H, Landgraf JM, Oostenbrink R, Moll HA, Essink-Bot ML. Reliability and validity of the infant and toddler quality of life questionnaire (ITQOL) in a general population and respiratory disease sample. Qual Life Res, Apr 2007. 16(3): p. 445-60.

R.T. Mangunkusumo, J. Brug, J. Duisterhout, H.J. de Koning, H. Raat, Feasibility, acceptability, and quality of Internet-administered adolescent health promotion in a preventive-care setting. Health Educ Res, Feb 2007. 22(1): p. 1-13.

R.T. Mangunkusumo, J. Brug, H.J. de Koning, J. van der Lei, H. Raat, School-based Internet-tailored fruit and vegetable education combined with brief counselling increases children's awareness of intake levels. Public Health Nutr, March 2007. 10(3): p. 273-9.

Bannink EMN, Raat H, Mulder PGH, Muinck Keizer-Schrama SMPF. Quality of life after growth hormone therapy and induced puberty in women with Turner syndrome. J Pediatr 2006;148(1):95-101.

Mohangoo AD, Linden MW van der, Schellevis FG, Raat H. Prevalence estimates of asthma or COPD from a health interview survey and from general practitioner registration: what's the difference? Eur J Public Health 2006;16(1):101-105.

Mangunkusumo RT, Duisterhout JS, Graaff N de, Maarsingh EJ, Koning H J de, Raat H. Internet versus paper mode of health and health behavior questionnaires in elementary schools: asthma and fruit as examples. J School Health 2006;76:80-86.

Beirens TM, Beeck EF van, Dekker R, Brug J, Raat H. Unsafe storage of poisons in homes with toddlers. Accid Anal Prev 2006;38(4):772-6.

Raat H, Bonsel GJ, Essink-Bot ML, Landgraf JM, Gemke RJ. Reliability and validity of comprehensive health status measures in children: The Child Health Questionnaire in relation to the Health Utilities Index. J Clin Epidemiol 2002;55(1):67-76.

Raat H, Bonsel GJ, Hoogeveen WC, Essink-Bot ML. Feasibility and reliability of a mailed questionnaire to obtain visual analogue scale valuations for health states defined by the Health Utilities Index Mark 3. Med Care 2004;42(1):13-8.

Bengi-Arslan, L., F. C. Verhulst, J. Ende, et al. (1997). "Understanding childhood (problem) behaviors from a cultural perspective: comparison of problem behaviors and competencies in Turkish immigrant, Turkish and Dutch children." Social Psychiatry and Psychiatric Epidemiology V32(8): 477-484.

Crijnen, A. A., T. M. Achenbach and F. C. Verhulst (1999). "Problems reported by parents of children in multiple cultures: the Child Behavior Checklist syndrome constructs." Am J Psychiatry 156(4): 569-74.

Jansen, W., A. van Berkel and N. van Veelen-Dieleman (2003). Rotterdamse Jeugdmonitor: groep 6 2000-2001. Rotterdam, GGD Rotterdam e.o., sector Jeugd.

Mackenbach, J. P. (2005). "Europe's precious children." Eur J Public Health 15(6): 555-556.

Muris, P., C. Meesters, et al. (2004). "The self-report version of the Strengths and Difficulties Questionnaire: its psychometric properties in 8- to 13-year-old non-clinical children." Br J Clin Psychol 43(Pt 4): 437-48.

Muris, P., C. Meesters, et al. (1995). "Samenhang tussen de Child Behavior Checklist (CBCL) en de DSM-classificatie." Kind en Adolescent 16: 170-175.

Muris, P., C. Meesters et al. (2003). "The Strengths and Difficulties Questionnaire (SDQ)--further evidence for its reliability and validity in a community sample of Dutch children and adolescents." Eur Child Adolesc Psychiatry 12(1): 1-8.

Verhulst, F., H. Koot et al. (1994). "Differential predictive value of parents' and teachers' reports of children's problem behaviors: A longitudinal study." Journal of Abnormal Child Psychology 22(5): 531-546.

Verhulst, F. C., T. M. Achenbach, et al. (2003). "Comparisons of Problems Reported by Youths From Seven Countries." Am J Psychiatry 160(8): 1479-1485.

Verhulst, F. C. et al. (1997). "Factors associated with child mental health service use in the community." J Am Acad Child Adolesc Psychiatry 36(7): 901-9.

RESULTS SYSTEMATIC REVIEW (#1-25)

[1] Briggs-Gowan M-J. A parent assessment of social-emotional and behavior problems and competence for infants and toddlers. Dissertation Abst Int B. 1996;57(6-B).

[2] Briggs-Gowan M-J et al. Preliminary acceptability and psychometrics of the Infant-Toddler Social and Emotional Assessment (ITSEA). Inf Ment Health J. 1998;19(4):422-45.

[3] Carter A-S et al. The infant-toddler social and emotional assessment (ITSEA). Inf Ment Health J. 1999;20(4):375-92.

[4] Maguire C. The prevention of developmental and behavioral problems of very preterm infants and parental stress through the use of developmental care. Leiden: ZonMw 2000.

[5] Briggs-Gowan MJ et al. Prevalence of social-emotional and behavioral problems in a community sample of 1- and 2-year-old children. J Am Acad Child Adolesc Psychiatry. 2001 Jul;40(7):811-9.

[6] Davis M-S. A comparison of three social emotional screening instruments. Dissertation Abst Int B. 2002;63(6-B).

[7] Carter AS et al. The Infant-Toddler Social and Emotional Assessment (ITSEA): factor structure, reliability, and validity. J Abnorm Child Psychol. 2003 Oct;31(5):495-514.

[8] Bracha Z et al. A French Adaptation of the Infant-Toddler Social and Emotional Assessment. Inf Ment Health J. 2004;25(2):117-29.

[9] Briggs-Gowan et al. The Brief Infant-Toddler Social and Emotional Assessment: screening for social-emotional problems. J Pediatr Psychol. 2004 Mar;29(2):143-55.

[10] Carter AS et al. Assessment of young children's social-emotional development and psychopathology. J Child Psychol Psychiatry. 2004 Jan;45(1):109-34.

[11] Ellingson KD et al. Parent identification of early emerging child behavior problems. Arch Pediatr Adolesc Med. 2004 Aug;158(8):766-72.

[12] Skovgaard AM et al. Assessment and classification of psychopathology in epidemiological research of children 0-3 years. Eur Child Adolesc Psychiatry. 2004 Dec;13(6):337-46.

[13] Briggs-Gowan MJ et al. Brief Infant Toddler Social and Emotional Assessment (BITSEA) Manual. New Haven: Yale University; 2005.

[14] Carter AS et al. Infant Toddler Social and Emotional Assessment (ITSEA) Manual. New Haven: Yale University; 2005.

[15] Zeijl E et al. Kinderen in Nederland. Den Haag: SCP, TNO; 2005.

[16] Briggs-Gowan MJ et al. Are infant-toddler social-emotional and behavioral problems transient? J Am Acad Child Adolesc Psychiatry. 2006 Jul;45(7):849-58.

[17] Toste C-P. Characteristics of exuberance. Dissertation Abst Int B. 2006;67(3-B).

[18] Barlow J et al. Role of home visiting in improving parenting and health in families at risk of abuse and neglect. Arch Dis Child. 2007 Mar;92(3):229-33.

[19] Bracha Z et al. Evaluation du developpement socioemotionnel de l'enfant d'un a trois ans. Neuropsychiatrie de l'Enfance et de l'Adolescence. 2007;55(4):194-203.

[20] Briggs-Gowan M-J et al. Applying the Infant-Toddler Social & Emotional Assessment (ITSEA) and Brief-ITSEA in early intervention. Inf Ment Health J. 2007;28(6):564-83.

[21] McDonald R et al. Violence toward a family member, angry adult conflict, and child adjustment difficulties. J Fam Psych. 2007;21(2):176-84.

[22] Theunissen M et al. Signaleringslijsten ter ondersteuning van het opsporen van psychosociale problemen bij kinderen van 0-4 jaar. Leiden: ZonMw, TNO 2007.

[23] Mittendorff M et al. In verwachting. Tilburg: ZonMw 2008.

[24] Radford P. A pilot study involving on asthma management program for inner-city early head start children. Phoenix Children's Hospital, Arizona, United States: AstraZeneca 2008.

[25] van-der-Pal S-M et al. Very pre-term infants' behaviour at 1 and 2 years of age and parental stress following basic developmental care. Brit J Dev Psych. 2008;26(1):103-15.

OVERVIEW OF STUDY POPULATIONS AND DESIGN
Validation of the BITSEA and evaluation of the BITSEA as screening tool in Youth Health Care for preschool children
H. Raat, MD, PhD, W. Jansen, MSc

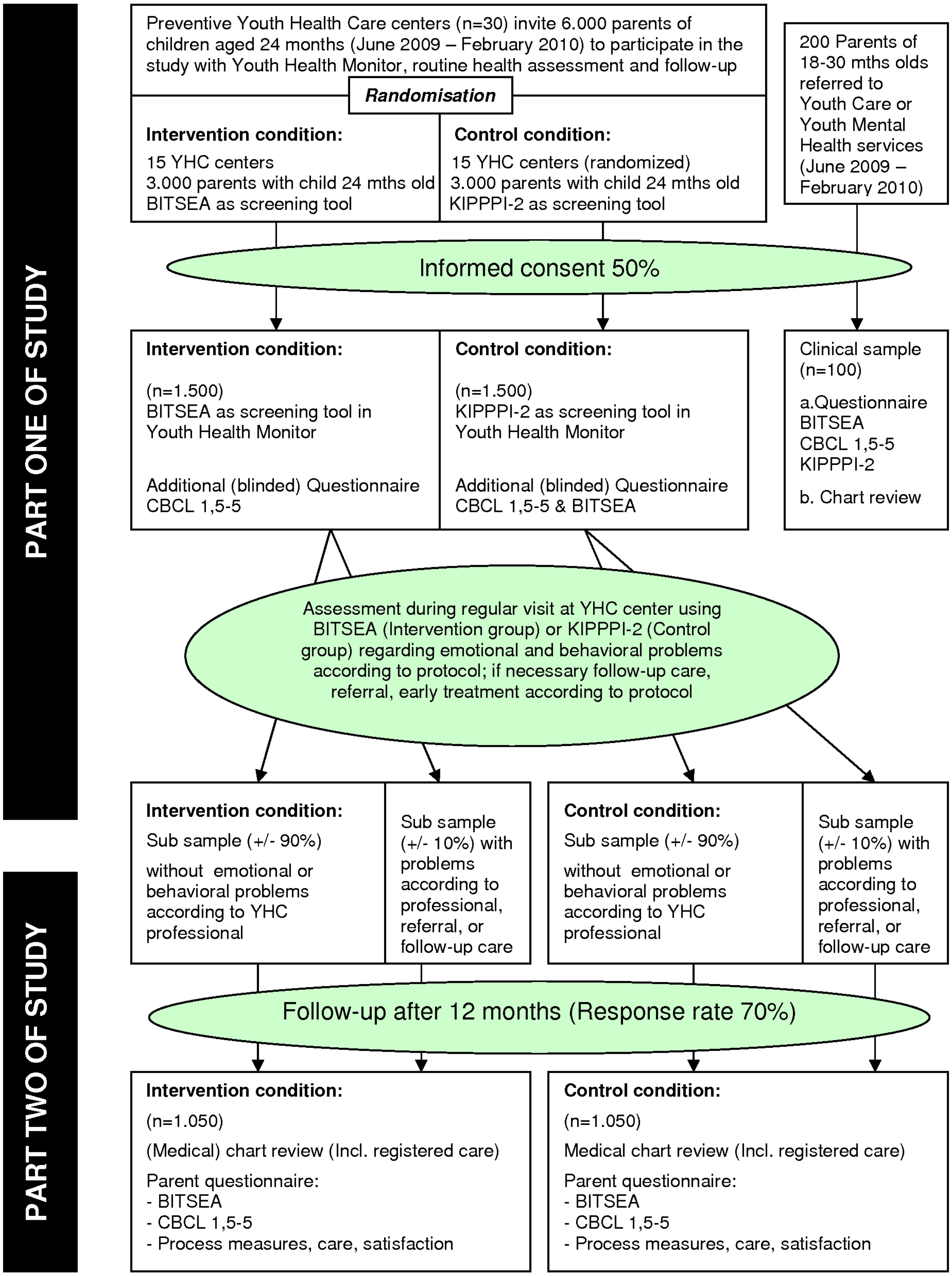

INCLUSION SCHEDULE PER MONTH (21 MONTHS)

Validation of the brief Infant-Toddler Social and Emotional Assessment (BITSEA) and evaluation of the BITSEA as screening tool in Youth Health Care for preschool children

H. Raat, MD,PhD, W. Jansen, MSc

	2009							2010											2011		
	J	J	A	S	O	N	D	J	F	M	A	M	J	J	A	S	O	N	D	J	F
Parents visiting preventive Youth Health Care Center with a child aged 24 months																					
**Inclusion of community sample (n= net 3000; 1500 intervention condition; 1500 control condition)**	5	10	20	40	60	80	100	125	160	200	200	200	200	200	200	200	200	200	200	200	200
Parents referred to or visiting Youth Care (Jeugdzorg) or Youth Mental Health Care with a child aged 18-30 months																					
**Inclusion of clinical sample (n= net 100)**	1	2	3	4	5	5	5	5	5	5	5	5	5	5	5	5	6	6	6	6	6

TIME SCHEDULE

Validation of the brief Infant-Toddler Social and Emotional Assessment (BITSEA) and evaluation of the BITSEA as screening tool in Youth Health Care for preschool children

H. Raat, MD,PhD, W. Jansen, MSc

	2008	2009												2010												2011												2012										
Task	D	J	F	M	A	M	J	J	A	S	O	N	D	J	F	M	A	M	J	J	A	S	O	N	D	J	F	M	A	M	J	J	A	S	O	N	D	J	F	M	A	M	J	J	A	S	O	N
PART ONE OF STUDY																																																
set up study *)	x	x	x	x	x	x																																										
training of child health professionals						x	x	x	x	x	x	x	x	x	x																																	
data collection community sample (YHC centers)							x	x	x	x	x	x	x	x	x	x	x	x	x	x	x	x	x	x	x	x	x																					
data collection clinical sample							x	x	x	x	x	x	x	x	x	x	x	x	x	x	x	x	x	x	x	x	x																					
data entry / cleaning part one study														x	x	x	x	x	x	x	x	x	x	x	x	x	x	x	x	x	x	x																
analyses part one study																	x	x	x	x	x	x	x	x	x	x	x	x	x	x	x	x	x	x	x	x	X											
preparing reports																										x	x	x	x	x	x	x	x	x	x	x	x	x	x	x	x	x	x	x	x	x	x	
PART TWO OF STUDY																																																
set up study *)	x	x	x	x	x	x																																										
training of child health professionals						x	x	x	x	x	x	x	x	x	x																																	
preparing follow up												x	x	x	x	x	x	x																														
data collection follow up																			x	x	x	x	x	x	x	x	x	x	x	x	x	x	x	x	x	x	x	x	x									
data entry / cleaning foll-up																								x	x	x	x	x	x	x	x	x	x	x	x	x	x	x	X	x	x							
analyses																											x	x	x	x	x	x	x	x	x	x	x	x	X	x	x	x	x	x	x			
preparing reports																																				x	x	x	x	x	x	x	x	x	x	x	x	
process interviews																x		x				x		x				x		x				x		x				x		x						
DISSEMINATION																																																
OF RESULTS																																																
Presentations												x												x						x						x						x						x
Expert meeting																																																x
Final report to ZonMw																																																x

*) preparing protocol, measures, receiving ethical approval, enrol partners for clinical sample, preparing data-entry, selection participating centers)
