# Supplementary material for: The Effectiveness of the BITSEA as a Tool to Early Detect Psychosocial Problems in Toddlers, a Cluster Randomized Trial
Source: PLoS One. 2015 Sep 18;10(9):e0136488. doi: 10.1371/journal.pone.0136488 (PMC4575038; doi:10.1371/journal.pone.0136488)
Supplement: S1 Table — (PDF) [file pone.0136488.s003.pdf]

S1 Table. Regression coefficients and confidence intervals (95% CI) from the multilevel regression models evaluating the association between condition and CBCL Total Problem score at follow-up corrected for confounders (n=2230).

	beta (95%CI)
**Primary outcome measure: CBCL Total Problem score at follow-up**	
Condition (intervention)	-2.32 (-3.75;-0.89)**
CBCL Total Problem score baseline	0.70 (0.66;0.74)***
Child gender (boy)	1.40 (0.34;2.48)**
Child ethnicity (native)	3.39 (-0.40;7.18)

NB. Correct for parental country of birth; parental educational level; age of the child; parental age.
*** p<0.001; **p<0.01
