# Supplementary material for: The Effectiveness of the BITSEA as a Tool to Early Detect Psychosocial Problems in Toddlers, a Cluster Randomized Trial
Source: PLoS One. 2015 Sep 18;10(9):e0136488. doi: 10.1371/journal.pone.0136488 (PMC4575038; doi:10.1371/journal.pone.0136488)
Supplement: S2 Table — (PDF) [file pone.0136488.s004.pdf]

S2 Table. **Number (percentages) of referred children in the total sample and in the subsample with baseline 'at risk' scores on the CBCL and BITSEA.**

	intervention		control		
**CBCL Total Problem score**	N	Referred	N	Referred	p
At risk score (score>60)	17 (1.41)	4 (23.5)	21 (1.50)	9 (42.9)	0.212
Low score	944 (78.2)	51 (5.4)	1149 (81.9)	81 (7.0)	0.123
**BITSEA Problem scale**					
At risk (score≥15 boys/ ≥14 girls)	89 (9.1)	9 (10.1)	n.a.	n.a.	n.a.
Low score	886 (90.9)	47 (5.3)	n.a.	n.a.	n.a.
**BITSEA Competence scale**					
At risk (score≤14 boys / ≤15 girls)	140 (14.3)	12 (8.6)	n.a.	n.a.	n.a.
Low score	839 (85.7)	44 (5.2)	n.a.	n.a.	n.a.
**Total**	983 (81.4)	56 (5.7)	1200 (85.5)	95 (7.9)	0.042*

* significant differences in number of referrals between intervention and control condition, as reported by child health professionals.
